# pH‐Dependent Effect of Lecithin on Lupin‐Protein‐Based Egg Custard Mimics

**DOI:** 10.1111/1750-3841.70479

**Published:** 2025-08-22

**Authors:** Uma Jingxin Tay, Jun Wei Ng, Shiyi Zhang, Daryl Lee, Marco Vignuzzi, Chengxin He, Dingsong Lin, Paolo Alberto Lorenzini, Maria N. Antipina, Weibiao Zhou, Dejian Huang

**Affiliations:** ^1^ Department of Food Science and Technology Bezos Centre for Sustainable Protein National University of Singapore Singapore Singapore; ^2^ Singapore Institute of Food and Biotechnology Innovation (SIFBI), Agency for Science Technology and Research (A*STAR) Singapore Singapore; ^3^ Infectious Disease Labs (ID Labs), Agency for Science Technology and Research (A*STAR) Singapore Singapore; ^4^ Bioinformatics Institute (BII), Agency for Science Technology and Research (A*STAR) Singapore Singapore; ^5^ Biomedical and Health Technology Innovation Platform National University of Singapore (Suzhou) Research Institute Suzhou Jiangsu China

## Abstract

**ABSTRACT:**

This study presents lupin‐protein‐stabilized emulsions across pH 6.0–8.0, containing 9.92% protein and 7.97% lipid, which formed egg custard mimics upon steaming. To support emulsion stability and potential nutritional benefits, soy lecithin, a phospholipid mixture, was added at 1.0% (w/w) to the emulsion. The effect of lecithin on the gel strength of the mimics was investigated, considering its varying impact across different protein systems. The binding affinity of phosphatidylcholine for β‐conglutin across pH 6.0–8.0 was consistent, ranging from −14.89 to −15.53 kcal/mol. At pH 8.0, hydrophobic interactions and hydrogen bonding among proteins were least extensive. Thus, those disrupted by phospholipid complexation were outweighed by those it promoted, both among proteins and between proteins and phospholipids. This resulted in smaller pores, reinforcing the gel strength of the mimic. In contrast, adding lecithin at pH 6.0 and 7.0 reduced hydrogen bonding, which at pH 6.0 also diminished disulfide bonding, ultimately weakening gel strength. As pH influenced protein retention through molecular interactions more strongly than lecithin, retention was highest at pH 6.0 (83.6%) and lowest at pH 8.0 (35.4%), both without lecithin. Correspondingly, these mimics were the strongest and weakest, respectively, based on dynamic consistency index (5.47 vs. 0.783 kPa s*ⁿ*) and breaking force (9.60 vs. 3.81 g). Overall, the mimics were perceived to have gel strength, moisture release, and greasiness comparable to egg custards, though they were grittier and stickier. Besides demonstrating their potential as nutritious egg alternatives, this study offers insight into the textural implications of protein gels incorporating lecithin.

**Practical Applications:**

This study presented plant‐based egg custard mimics made from lupin‐protein‐stabilized emulsions, which exhibited textures similar to traditional egg custards. Adding 1.0% soy lecithin altered gel strength in a pH‐dependent manner and slightly increased grittiness. These findings on how lecithin affected the texture of lupin‐protein emulsion gels provide food companies with practical insights for utilizing lecithin to enhance shelf stability and nutritional benefits in plant‐based products.

## 1 Introduction

Plant‐based egg products capable of forming the homogeneous and cohesive gels characteristic of egg custards could help drive demand in this category, which currently represents just 0.4% of US retail sales (Bushnell et al. 2024). In addition to being a more sustainable and ethical alternative, such products could meet the needs of individuals with egg allergies (Khalifa et al. 2025). To enable gelation upon heating in commercial plant‐based eggs, legume protein extracts are commonly used because they are rich in globulins, which are globular proteins similar to those in eggs (Wang et al. 2022; Lu et al. 2023). In‐line with this, the present study developed egg custard mimics using protein extracted from the legume *Lupinus albus*. Being similarly globulin‐rich, *L. albus* protein reportedly gelled at concentrations between 7% and 20% (Al‐Ali et al. 2021). The minimum gelling concentration and resulting gel strength varied depending on cultivation conditions and processing methods. With globulins comprising 87% of the protein content and the seeds containing 32%–35% protein on a dry basis (Al‐Ali et al. 2021), substantial protein extraction yields were expected (Muranyi et al. 2016), supporting the potential commercialization of the mimics. The yellowness of the extract, combined with the common consumption of *L. albus* seeds for their mild flavor profile (Duranti et al. 2008), further motivated this initial investigation into its suitability for developing an egg custard mimic.

Insoluble proteins may lead to grittiness (Grygorczyk and Blake 2023). Moreover, their tendency to sediment compromises the pourability of dispersions and the homogeneity of the resulting gels (Khalifa et al. 2025). Therefore, gels prepared using a higher soluble protein fraction were postulated to better mimic the texture of egg custard. Achieving such gels required the maintenance of high protein solubility. Spray drying, a common protein processing method, reportedly compromises solubility due to thermal and shear‐induced denaturation. Notably, the solubility of *L. albus* proteins at pH 8.0 was 85% when freeze‐dried but declined to 72% when spray‐dried (Devkota et al. 2023). Although freeze‐drying better preserves solubility, its limited scalability poses challenges for broader application. Thus, using the extracted proteins without drying appeared to be a relatively viable approach for preserving protein solubility.

Soy lecithin, which comprises a mixture of phospholipids, is commonly incorporated into legume protein‐stabilized emulsions to confer shelf stability. This functionality is attributed to the adsorption of soy phospholipids at oil droplet surfaces, both as monomers and in complexes with proteins, which reduces flocculation and coalescence (Son et al. 2025; Xia et al. 2024). Additionally, lecithin is a naturally occurring component in eggs. Thus, soy lecithin may help the mimic offer similar nutritional benefits, such as supporting cardiovascular, liver, and brain health (Caudill 2010; Hedayati and Tehrani 2018; Tay et al. 2025). The only known study examining the addition of soy lecithin to a plant protein emulsion gel was based on amaranth protein (Tay et al. 2025). Although phospholipid–protein complexes formed in the continuous phase were reported to alter protein interactions and strengthen the gel in this study, the incorporation of lecithin in other studies on porcine myofibrillar and surimi gels yielded differing effects on gel strength (Shi et al. 2020; Zhou et al. 2020; Xia et al. 2018). From these studies, it may be inferred that for lecithin to strengthen the gel, the hydrophobic interactions and hydrogen bonds promoted by its phospholipids—both between proteins and phospholipids, and among proteins via associated conformational changes—must outweigh those that are disrupted. Increases in these non‐covalent interactions were often accompanied by enhanced disulfide bonding, as they brought sulfhydryl groups into closer proximity and facilitated their oxidation into disulfide bonds during heating. Together, these interactions enhanced network connectivity and gel strength.

As lecithin phospholipids have been reported to complex with several legume proteins (Son et al. 2025; Xia et al. 2024; Xiao et al. 2024), they are likely to complex with lupin proteins as well. Therefore, the primary research question was how varying the pH of the mimics across pH 6.0–8.0 affected lecithin's modulation of gel strength. This pH range was selected on the basis of studies of soy protein gels, which share a similar isoelectric point (pH 4.5–5.5) (Duranti et al. 2008; Liang et al. 2024). Liang et al. (2024) reported that reduced electrostatic repulsion at pH 6.0 maximized protein–protein interactions during heating, without decreasing solubility to the extent that it inhibited gelation at pH 5.5, thereby enhancing gel strength. As the pH of the mimics increased, electrostatic repulsion was generally expected to increase, weakening hydrophobic interactions and hydrogen bonding among lupin proteins (Al‐Ali et al. 2021). Thus, when lecithin was added at pH 8.0, it was hypothesized that the hydrophobic interactions and hydrogen bonds formed between proteins and phospholipids, as well as among proteins, outweighed those that were disrupted. This likely promoted disulfide bonding, improved network connectivity, and enhanced gel strength. In contrast, the opposite trend was expected at pH 6.0 and 7.0. The hypothesis was evaluated through a novel characterization of pH‐dependent phospholipid–protein complexation. Specifically, the surfaces of the dominant lupin proteins, identified via SDS–PAGE, were modeled at pH 6.0, 7.0, and 8.0 for deep learning‐based docking with soy phosphatidylcholine. Phospholipid–protein complexation was linked to lecithin‐induced changes in protein aggregation, intermolecular interactions, and microstructure, which together explained the observed differences in gel strength among the mimics.

This led to a secondary question concerning how lecithin influenced the perceived texture of the mimics and whether the texture of the lecithin‐containing mimics was within the typical range observed for egg custards. Quantitative descriptive analysis (QDA) was used to address this question, contributing to knowledge generation. The lupin‐protein emulsions could also be applied in products that rely on egg‐induced binding and texturization. More broadly, this is the first known investigation to characterize how lecithin influences the strength of protein emulsion gels across a range of pH values. This is particularly notable given soy lecithin's widespread use to enhance emulsion stability, owing to its cost‐effectiveness as a by‐product of soybean oil refining (Pan et al. 2024). As protein emulsion gels often serve as the structural base for plant‐based egg, meat, and seafood alternatives, these novel insights could inform the development of such products to meet growing consumer demand (Bushnell et al. 2024).

## 2 Materials and Methods

### 2.1 Materials

Lupin flour (*L. albus*) was purchased from Lupins for Life (New South Wales, Australia). Chicken eggs and canola oil were purchased from NTUC Fairprice Co‐operative (Singapore). Soy lecithin was purchased from NaturesPlus (Natural Organics Inc., Melville, New York, USA). Myvegan Soy Protein Isolate was purchased from The Hut Group (United Kingdom). Plant‐based eggs purchased include Hegg Eggless Egg from Hegg Foods (Singapore) and Savoury Egg Mix from Plantasy Foods (Australia). SDS–PAGE reagents were purchased from Bio‐Rad Laboratories (Hercules, California, United States). All other chemicals used were of analytical grade and purchased from Merck KGaA (Darmstadt, Germany).

### 2.2 Sample Preparation

#### 2.2.1 Preparation of Lupin‐Protein Supernatant Lupin‐Protein Supernatant 1 (LS1)

Lupin flour was defatted using a 1:10 flour‐to‐hexane ratio through two extraction cycles, each lasting 10 h. The defatted flour, obtained via vacuum filtration, was air‐dried at 25°C for at least 36 h. Subsequently, it was dispersed at 10% w/v in deionized water, and the pH was adjusted to 9.0 ± 0.05 using 1.0 M NaOH to solubilize the proteins. The dispersion was centrifuged twice at 12,857 × *g* for 10 min. The supernatant was acidified to pH 4.50 ± 0.05 using 1.0 M HCl and centrifuged under the same conditions. The resulting protein precipitate was resuspended and adjusted to pH 8.0 ± 0.05 in deionized water using 1.0 M NaOH by homogenizing with an Ultra‐Turrax T25 homogenizer (IKA, Staufen, Germany) to form the extract. The solid content of the extract, determined using AOAC Method 930.15, was typically 20%–25% w/w. Therefore, the extract was diluted to solid contents of 15.0%–20.0% at 2.5% intervals. To facilitate dilution, the extract was homogenized at 10,000 rpm for 30 s with deionized water (Ultra‐Turrax T25), while maintaining the pH at 8.0 ± 0.05 using 0.1 M NaOH. The dispersion was then left to hydrate for 45 min before centrifugation at 12,857 × *g* for 10 min to yield a supernatant. The minimum solid content required to produce a supernatant capable of gelation upon steaming—referred to as LS1—was 17.5%.

#### 2.2.2 Preparation of Control Egg Custard and Egg Custard Mimics

The control egg custard, labeled “C,” was prepared by diluting whole hen egg to 45% (w/w) to form the control liquid egg (Wang et al. 2022), followed by steaming for 10 min. To prepare the egg custard mimics, the original pH of the LS1 supernatant (8.0 ± 0.05) was adjusted to 7.0 and 6.0 ± 0.05 using 1.0 M HCl during homogenization at 10,000 rpm with an Ultra‐Turrax T25. After pH adjustment, the solid content of each dispersion was determined using AOAC Method 930.15. The LS1 dispersions typically contained 14%–15% (w/w) total solids, corresponding to approximately 11%–12% (w/w) protein, based on the protein purity of LS1 (76.2%) as determined by the method described in Section 2.3 and presented in Table S1. Accordingly, emulsions could be prepared at a maximum of 80% (w/w) egg equivalent, corresponding to 9.92% (w/w) protein and 7.97% (w/w) lipid. Given that the control liquid egg contained 45% egg (w/w), emulsions with 30%–80% egg‐equivalent content (w/w)—formulated at 10% intervals based on the protein and lipid contents of egg—were evaluated (Table S2). Deionized water was added to the LS1 dispersions at the respective pH, based on the measured solid content, to achieve the target protein concentration. The pH was maintained during dilution by adding 0.1 M NaOH and/or 0.1 M HCl as needed. The resulting dispersions were then emulsified with 3.0%–7.9% w/w canola oil for 30 s at 10,000 rpm using an Ultra‐Turrax T25 (Table S2). Emulsions that did not flow upon inversion after 10 min of steaming were considered gelled.

The lowest concentration at which the emulsion gelled at pH 8.0 was 80% (w/w) egg equivalent, whereas emulsions at pH 6.0 and 7.0 gelled at concentrations as low as 70% (w/w) egg equivalent (Figure S1). Therefore, egg custard mimics were prepared from emulsions across pH 6.0–8.0 at 80% (w/w) egg equivalent, each containing 7.9% (w/w) oil. For the lecithin‐containing mimics, soy lecithin was incorporated at 1.0% w/w of the emulsion by adding it to the diluted LS1 dispersions at the respective pH (1.08% w/w) and hydrating for 30 min prior to emulsification and steaming. Mimics prepared from emulsions containing lecithin are denoted by a (+) suffix on their pH value, whereas those without lecithin are denoted by a (−) suffix. Table S3 presents the ingredient and proximate compositions of the control liquid egg and the emulsions selected for preparing the mimics.

### 2.3 Proximate Analysis of the Ingredients

LS1, soy lecithin, and hen egg were dried using AOAC Method 930.15 for the subsequent analyses (AOAC 2023). Their ash content was determined using AOAC Method 923.03 (AOAC 2023). Their nitrogen content was determined using the Dumas method with a FlashSmart CHNS Elemental Analyzer (Thermo Fisher Scientific), following AOAC Method 968.06 (AOAC 2023). The nitrogen‐to‐protein conversion factors applied were 5.44 for LS1, as it is composed of lupin protein, and 6.25 for egg. (Mariotti et al. 2008). Their lipid content was measured using the Soxtherm Extraction System (C. Gerhardt Analytical Systems, Konigswinter, Germany) following AOCS Procedure Am 5‐04 (American Oil Chemists' Society 2017). The proximate composition of LS1 was expressed on a dry basis due to its variable moisture content, as it was the supernatant of the lupin‐protein extract obtained without drying. Thus, carbohydrate content of LS1 was calculated by subtracting the sum of ash, protein, and lipid contents from 100%. For the hen egg, measured on a wet basis, carbohydrate content was determined by additionally subtracting the moisture content.

### 2.4 SDS–PAGE Analysis of Protein Composition in the Supernatant and Pellet

The protein composition of LS1 was analyzed in comparison to that of its corresponding pellet, referred to as lupin‐protein pellet 1 (LP1). LP1 was dispersed to 17.5% w/w solids and centrifuged at 12,857 × *g* for 10 min to yield lupin‐protein supernatant 2 (LS2) and pellet 2 (LP2), following the procedure used to prepare LS1 and LP1 from the lupin‐protein extract. LS1, LP1, LS2, and LP2 were each diluted to 2.0 mg protein/mL, as determined by the Bradford assay, where absorbance at 595 nm was measured against a bovine serum albumin standard curve (62.5–1000 µg/mL) using a microplate spectrophotometer (Synergy HT, BioTek Instruments, Winooski, VT, USA). Each diluted sample was mixed with 4× Laemmli buffer supplemented with 2.5% (v/v) β‐mercaptoethanol (βME) for the reducing condition. After heating at 95°C for 10 min, the samples were loaded onto a polyacrylamide gel (5% stacking and 10% separating), which was run using the Mini‐PROTEAN 3 electrophoresis cell unit (Bio‐Rad, Hercules, CA, USA). Molecular weights and band intensities were analyzed using Image Lab 6.1.0 software (Bio‐Rad), with band intensities normalized to the 50 kDa band on the molecular weight ladder.

### 2.5 β‐Conglutin Modeling and Soy Phosphatidylcholine Docking

The structure of soy phosphatidylcholine (C_42_H_80_NO_8_P) was downloaded from the Chemical Compound Deep Data Source database (https://www.molinstincts.com/). The structure of β‐conglutin from *L. albus*, determined by x‐ray diffraction, was obtained from the Protein Data Bank (PDB ID: 8OFD). Protonation states were assigned at pH 6.0, 7.0, and 8.0 using the “PlayMolecule Protein Prepare” tool (Martínez‐Rosell et al. 2017). Using an NVIDIA A100‐40G SXM GPU, blind flexible docking was performed by inputting the whole protein coordinates into GNINA 1.0 (McNutt et al. 2021), with “num_poses = 9” and “exhaustiveness = 220.” The docking results were visualized using UCSF ChimeraX and Flare (Kuhn et al. 2020).

### 2.6 Characterization of Protein Dispersions Used to Prepare the Mimics

LS1 dispersions at pH 8.0, 7.0, and 6.0 ± 0.05, each containing 11.5% w/w protein, were formulated with and without 1.08% soy lecithin, as described in Section 2.2. Achievable soluble protein content was determined by centrifuging the dispersions at 12,857 × *g* for 10 min, matching the conditions used in LS1 preparation from the lupin‐protein extract. The total solids content in the resulting supernatants was determined using AOAC Method 930.15 and converted to protein content based on the protein purity of LS1 (76.2%), as described in Section 2.3. For particle size and zeta potential measurements, the dispersions were diluted 100‐fold with deionized water, and the pH was adjusted to match the original values of each dispersion using 0.1 M NaOH or 0.1 M HCl. Particle size was measured using a Zetasizer Nano ZS (Malvern Panalytical, Malvern, Worcestershire, United Kingdom) (Pan et al. 2024). Zeta potential was determined using a NanoBrook Omni particle size and zeta potential analyzer (Brookhaven Instruments, Holtsville, New York, USA) operated in phase analysis light scattering (PALS) mode, with the Smoluchowski model applied.

### 2.7 Molecular Interactions in the Mimics

Molecular interactions were assessed by dispersing 2.0 ± 0.1 g of mimics in 10.0 mL of Solvent A–E at 10,000 rpm for 1 min (Ultra‐Turrax T25), followed by incubation for 45 min and centrifugation at 12,857 × *g* for 10 min. Solvent A was deionized water; Solvent B was 0.6 M NaCl in 0.1 M potassium phosphate buffer at pH 6.0, 7.0, and 8.0 for the corresponding mimics; Solvent C was Solvent B with 1.5 M urea; Solvent D was Solvent B with 8 M urea; and Solvent E was Solvent D with 0.5 M βME. The protein content in each supernatant was measured using the Bradford assay. Protein solubilized in Solvent A was considered unbound. Differences in the amount of protein solubilized between Solvents A and B, B and C, C and D, and D and E corresponded to the amount stabilized by ionic bonds, hydrogen bonds, hydrophobic interactions, and disulfide bonds, respectively (Tay et al. 2025). These amounts were expressed as a proportion of the total protein content in the mimic, approximately 198 mg.

### 2.8 Scanning Electron Microscopy (SEM) of the Mimics

Freeze‐dried egg custard mimics were platinum sputter‐coated using a JFC‐1600 Auto Fine Coater (Jeol Ltd., Tokyo, Japan) and imaged at 15 kV and 1500× magnification with an SEM microscope (JSM‐6701F, Jeol Ltd.).

### 2.9 Oil Droplet Volume Analysis in the Mimics Using Confocal Laser Scanning Microscope (CLSM)

Slices of 5 mm × 5 mm × 1 mm of the egg custard mimics were immersed for 15 min in an ethanol solution containing 29 ppm fluorescein‐5‐isothiocyanate (FITC) to label the protein and 170 ppm Nile red to label the oil droplets. After rinsing for 5 min in a 50% v/v solution, imaging was performed at 100× magnification using a CLSM (LSM 710, Carl Zeiss Inc., Oberkochen, Germany) operated with ZEN software (Carl Zeiss Inc.). The excitation/emission wavelengths for FITC were 488/497–553 nm, and for Nile red were 543/553–693 nm. Three‐dimensional (3D) images were reconstructed from z‐stacks, and oil droplets were modeled for volume calculation using Imaris (Bitplane, Belfast, United Kingdom).

### 2.10 Analytical Gel Strength of the Mimics

#### 2.10.1 Breaking Force

A TA‐XT2i texture analyzer (Stable Micro Systems, Surrey, UK) with a P25 probe was used to measure the breaking force. The measurement was performed with a penetration distance of 8 mm, a pre‐test speed of 1.0 mm/s, a test speed of 0.5 mm/s, a post‐test speed of 10 mm/s, and a trigger force of 0.1 g.

#### 2.10.2 Frequency Sweep

An MCR 102 rheometer (Anton Paar, Graz, Austria) connected to RheoCompass Software Version 1.33.54 (Anton Paar) and fitted with a 25 mm parallel plate at a plate‐sample gap of 500 µm was used. The storage (*G′*) and loss (*G″*) moduli of the control egg custard and the mimics were measured as a function of angular frequency (*ω*) from 0.335 to 100 rad/s at 0.5% strain within the linear viscoelastic region. Using Prism 10 (GraphPad, California, USA), *G′* and *G″* were fitted to power law models (Equations 1 and 2) and used to compute complex viscosity η*, which was then fitted to the Ostwald‐de Waele model (Equation 3) (Alghooneh et al. 2017):

(1)

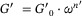



(2)

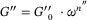

where *G′_0_* (Pa s*^n′^*) and *G″*_0_ (Pa s*^n″^*) represent *G′* and *G″* at 1 rad/s, respectively, whereas *n′* and *n″* indicate their frequency dependence.

(3) $$\eta^{*}=K_{f}\cdot \omega^{n_{f}-1}$$

where *K_f_* referred to the dynamic consistency index (Pa $s^{n_{f}}$) and *n_f_* to the flow behavior index.

### 2.11 QDA of the Mimics

#### 2.11.1 Sensory Panel

The National University of Singapore Institutional Review Board approved the exemption of the QDA from review (NUS‐IRB‐2023‐291) on May 25, 2023. All subjects provided informed consent and confirmed the absence of allergies to lupin, soy, pea, or egg proteins.

#### 2.11.2 Procedure

Screening tests and sample evaluations were conducted in individual booths under white light at 22°C, with responses recorded using Compusense (Ontario, Canada). Room‐temperature water was provided for palate cleansing during a 1‐min rest—timed using an integrated stopwatch—between attributes in the screening test and between samples during evaluation. Texture attributes used for the sensory evaluation of emulsions (Fuhrmann et al. 2019) and custard puddings (Sasaki et al. 2019) were adopted for screening and guided panel discussions. The screening standards and intensity references were prepared from dispersions steamed for 10 min in sensory cups labeled with three‐digit blind codes, each containing 10 ± 0.5 g, and served within 5 min after steaming, as described in the following paragraphs. The proximate compositions of Savoury Egg Mix and Myvegan Soy Protein Isolate used in these dispersions were determined as described in Section 2.2.2 and are presented in Table S1. Nitrogen‐to‐protein conversion factors of 5.4 and 5.5 were used for Savoury Egg Mix (containing lupin and pea proteins) and Myvegan Soy Protein Isolate, respectively.

Participants aged 21–53 were screened for their sensitivity to “firmness,” “stickiness,” and “grittiness,” as defined in Table S2. For firmness, the set consisted of five egg custards prepared by steaming eggs diluted to 40%–60% w/w at 5% intervals. For grittiness, the set consisted of six egg custards prepared by steaming variants of the control liquid egg, each containing up to 5% w/w Myvegan Soy Protein Isolate in 1% increments. For stickiness, six hybrid egg custards were prepared by steaming mixtures of the control liquid egg combined at 40%–65% w/w with a dispersion containing 26.5% w/w Savoury Egg Mix powder in 5% increments. Participants were briefed on the attribute definitions using the extremes of the screening sets as references. They were then provided with screening sets for each attribute and instructed to sequence the samples within each set in order of increasing intensity. Among the 72 individuals screened, 17 participants (8 males and 9 females, aged 21–53) with the highest number of standards correctly sequenced across various attributes were recruited.

During panel discussions, the panel developed textural attributes to describe the mimics in relation to the control egg custard. Using a continuous 7‐point scale, where “1” represented the mildest and “7” the strongest intensity, they assigned ratings to various intensity references (Table S4). “Structural retention” referred to the gel's integrity before mastication, complementing “firmness,” which described resistance during mastication. Together, these attributes characterized gel strength, with lower and higher intensities evaluated relative to the control egg custard, which was anchored using variants prepared by steaming whole hen eggs diluted to 30% and 80% (w/w), respectively. A lower intensity of “moisture release” and a higher intensity of “stickiness,” relative to the control egg custard, were anchored using a gel prepared by steaming a dispersion of 26.4% (w/w) Savoury Egg Mix in deionized water (Table S3). Conversely, a higher intensity of “moisture release” was anchored by a particulate dispersion prepared by steaming egg diluted to 15% w/w. A higher intensity of “greasiness” relative to the control egg custard was anchored using an oil‐added variant, prepared by homogenizing 20.0% w/w canola oil into the control liquid egg at 8000 rpm for 30 s (L4RT high‐shear mixer, Silverson Machines, United Kingdom), followed by steaming (Table S3). Conversely, a lack of “greasiness” and a higher intensity of “grittiness,” relative to the control egg custard, were anchored by a gel prepared by steaming an emulsion of Myvegan Soy Protein Isolate, which contained 9.9% protein and 8.0% lipid, similar to those of the emulsions used to prepare the egg custard mimics. The emulsion was prepared by dispersing Myvegan Soy Protein Isolate, with a protein purity of 73.9% (Table S1), at 14.5% w/w in deionized water, which was homogenized with canola oil at 7.90% w/w at 8000 rpm for 2 min (L4RT high‐shear mixer) (Table S3). To prepare intermediate‐intensity references for “stickiness” and “grittiness,” plant‐based dispersions were blended with the control liquid egg in varying proportions at 8000 rpm for 2 min (L4RT high‐shear mixer, Silverson Machines, United Kingdom), as detailed in Table S2, and then steamed. During evaluation, panelists individually scored the intensity of each attribute on its anchored continuous 7‐point scale, following the sequence in Table S4. The egg custard mimics were presented in a randomized monadic order, with each panelist evaluating them in triplicate, one replicate per session (Morais Ferreira et al. 2017).

### 2.12 Statistical Analysis

Experiments were performed in biological triplicates unless stated otherwise. Results were expressed as mean ± standard deviation. Statistical and graphical analyses were conducted using Prism 9.4.1 (GraphPad Software LLC, California, USA). To determine significant differences (*p* < 0.05), one‐way ANOVA followed by Tukey's HSD test was used to compare the means **among** the control egg custard and the mimics, whereas unpaired *t*‐tests were used for comparisons within each sample.

## 3 Results and Discussion

### 3.1 Preparation and Appearance of Egg Custard Mimics

The protein extraction yield from lupin flour was 51.70% ± 1.56% for the lupin‐protein extract and 10.80% ± 2.07% for the LS1 derived from it. Thus, using LS1 to prepare the mimics substantially reduced the extraction yield. This occurred despite LS1 being prepared at the minimum solid content required to obtain a supernatant capable of gelation upon steaming—a condition that maximized supernatant volume and, in turn, the amount of protein solubilized. Egg custard mimics were prepared from LS1‐based emulsions across pH 6.0–8.0 at 80% (w/w) egg equivalent, corresponding to 9.92% protein and 7.97% lipid. This represented not only the highest achievable concentration based on the protein content of LS1 dispersions but also the lowest concentration at which the emulsion gelled at pH 8.0 (Section 2.2.2). By contrast, emulsions at pH 6.0 and 7.0 gelled at concentrations as low as 70% (w/w) egg equivalent (Figure S1), likely due to reduced electrostatic repulsion facilitating protein aggregation (Liang et al. 2024).

**FIGURE 1:**
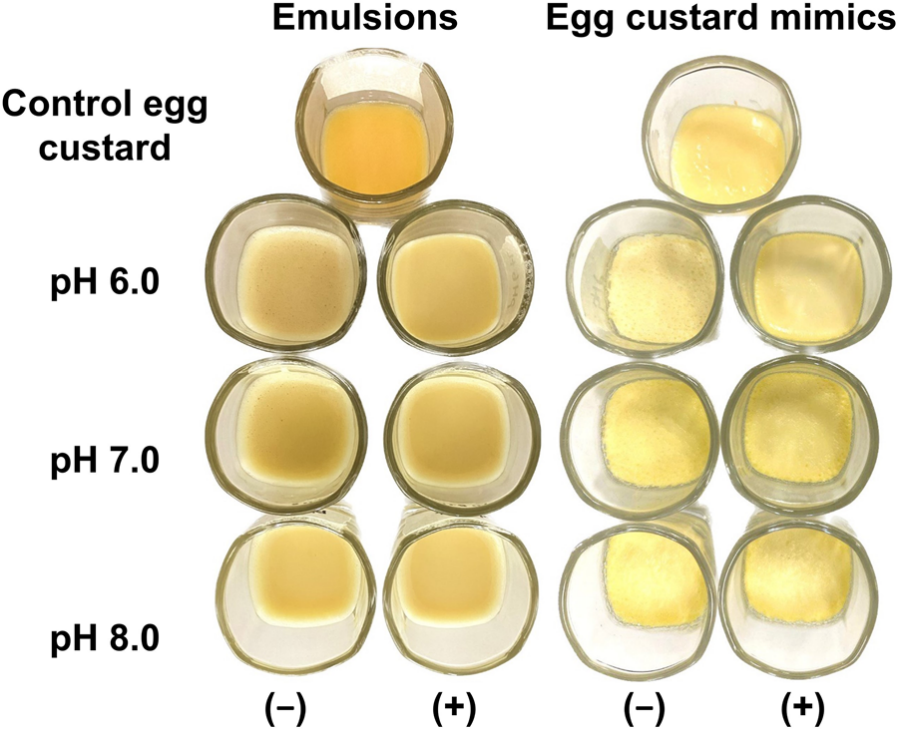
Appearance of unheated emulsions and the corresponding egg custard mimics formed upon steaming at different pH levels. (−) indicates the absence and (+) the presence of lecithin, shown relative to the control liquid egg and the corresponding control egg custard.

Soy lecithin was incorporated at 1.0% w/w of the emulsion, as this reportedly provides sufficient phospholipids to coat oil droplet surfaces, thereby supporting emulsion shelf stability (Son et al. 2025; Xia et al. 2024). Additionally, this concentration is within the range of lecithin content found in eggs (1.0%–3.0% w/w), supporting the mimics in offering similar nutritional benefits (Caudill 2010; Hedayati and Tehrani 2018; Tay et al. 2025). Overall, the mimics closely resembled the control egg custard in appearance (Figure 1). Emulsions lacking lecithin also formed homogeneous gels without phase separation, as they were steamed within 1 h of preparation to maintain focus on the effect of pH on lecithin's modulation of gel strength.

### 3.2 Analysis of Protein Composition in the Supernatant and Pellet via SDS–PAGE

**FIGURE 2:**
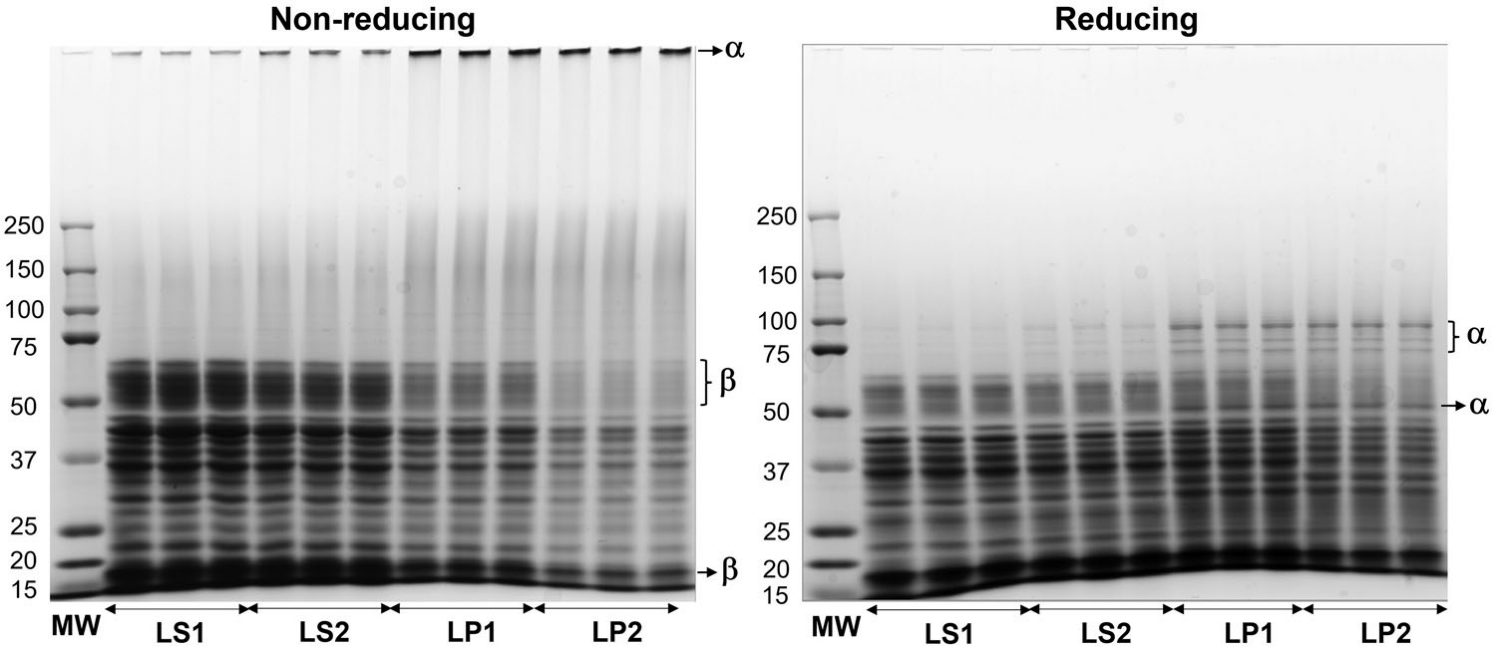
SDS–PAGE profiles of LS1 compared to LS2, LP1, and LP2 under non‐reducing and reducing conditions. Proteins responsible for changes in band patterns are annotated, with β indicating β‐conglutin and α indicating α‐conglutin. LP1, lupin protein pellet 1; LS1, lupin protein supernatant 1.

The protein composition of LS1 was compared to that of LP1, its corresponding pellet. To substantiate the tendency of the respective proteins to partition between the supernatant and pellet phases, LP1 was redispersed and centrifuged, yielding LS2 and LP2. Under non‐reducing conditions, the cumulative intensity of bands in the range of 50.97 ± 0.42–63.50 ± 0.26 kDa gradually decreased (Figure 2), from 0.47 for LS1 to 0.26 for LS2, 0.16 for LP1, and finally 0.04 for LP2 (*p* < 0.05) (Table S5). Similarly, under reducing conditions, the cumulative intensity of these bands (52.65 ± 0.75–64.15 ± 1.25 kDa) progressively decreased in the same order (*p* < 0.05). The retention of the 51.0–64.2 kDa bands under both reducing and non‐reducing conditions indicated the absence of disulfide linkages in the proteins associated with these bands, a characteristic feature of β‐conglutin. This distinguishes it from α‐conglutin, the other major lupin globulin, whose intact form and acidic subunit also produce bands in this range (Duranti et al. 2008). This indicated that β‐conglutin was gradually depleted from LS1 to LS2, LP1, and ultimately LP2.

Under non‐reducing conditions, the intensity of the band retained in the well increased approximately 1.5‐fold from LS1 to LS2 and again from LS2 to LP1 and LP2 (*p* < 0.05) (Figure 2, Table S5). Upon reduction, this band disappeared—with intensities falling below 0.02 across all samples—and multiple new bands emerged, showing increasing intensities in the same order. A 52.30 ± 0.38 kDa band appeared in LP1 and LP2 (*p* < 0.05). Additionally, 75.40 ± 0.41, 83.65 ± 0.52, and 96.55 ± 0.62 kDa bands were detected in LS2, with their cumulative intensity being higher in LP1 and LP2 (*p* < 0.05) (Table S5). The band entrapped in the wells had a molecular weight exceeding 250 kDa. Its presence only under non‐reducing conditions indicated that it was composed of α‐conglutin, which typically aggregates into a hexamer of 330–430 kDa (Duranti et al. 2008). Reducing the disulfide bonds in α‐conglutin hexamers likely released the acidic subunit, which could have contributed to the 52.30 kDa band. However, incomplete reduction likely caused some α‐conglutin to remain intact, resulting in the 75.4 kDa band. Additionally, interactions between intact α‐conglutin and the acidic and basic subunits might have led to the formation of higher molecular weight bands at 83.7 and 96.6 kDa (Duranti et al. 2008; Fontanari et al. 2011). The increasing prominence of the α‐conglutin hexamer from LS1 to LS2 to LP1 and LP2 under non‐reducing conditions, along with the growing intensity of its fragments under reducing conditions, indicated that α‐conglutin was lost to the pellets. Correspondingly, β‐conglutin was enriched in the supernatants, particularly in LS1 relative to the whole protein extract, consistent with reports of the higher solubility of 7S globulin (β‐conglutin) compared to 11S globulin (α‐conglutin) across various legume proteins (Quiroga et al. 2012; Johansson et al. 2023). This, coupled with β‐conglutin generally constituting a higher proportion of *L. albus* globulins (54%–44%) compared to α‐conglutin (37%–31%) (Duranti et al. 2008; Czubinski and Feder 2019), led to its selection for molecular docking.

### 3.3 β‐Conglutin Modeling and Soy Phosphatidylcholine Docking

**FIGURE 3:**
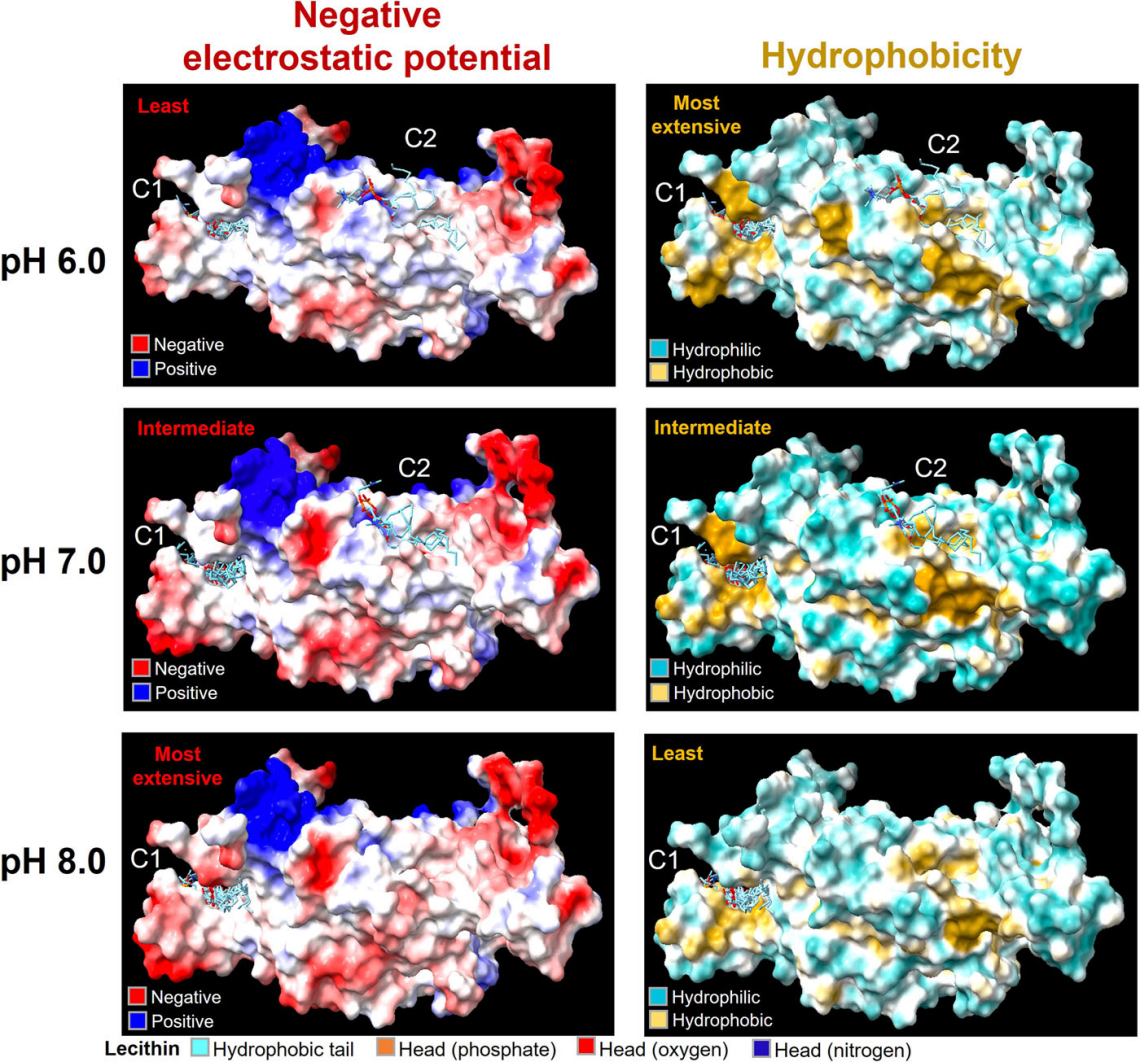
Surface properties of β‐conglutin and the docking of phosphatidylcholine at different pH levels. C1 denotes Cavity 1, and C2 denotes Cavity 2. The back view is shown in Figure S2.

As the pH increased from 6.0 to 8.0, amino acid deprotonation caused neutral regions (white) on the β‐conglutin surface to shift towards negative (red), whereas positive regions (blue) shifted slightly towards neutral (Figure 3 and Figure S2). Regions that became more negatively charged exhibited a decrease in yellow (indicative of hydrophobicity), with a corresponding increase in blue (hydrophilicity) and white (intermediate polarity). At pH 6.0 and 7.0, two soy phosphatidylcholine molecules were predicted to dock in Cavity 2 of β‐conglutin, whereas the remaining seven docked in Cavity 1. However, at pH 8.0, all poses were confined to Cavity 1. Affinity of the top poses was similar across the cavities and pH values, ranging from −14.89 to −15.53 kcal/mol for binding affinity and from 5.63 to 5.96 for CNN affinity (Table S6). The phosphorus and oxygen atoms in the top pose hydrogen bonded with Arg404, Arg422, Gly468, and Gln500 in Cavity 1, along with Gln123, Gln141, Arg153, and Phe238 in Cavity 2 (Figure S3). Additionally, the oxygen atom electrostatically interacted with Arg404 in Cavity 1. The alkane tails hydrophobically interacted with Pro379, Glu399, Val401, Ile403, Val420, Arg422, Tyr439, Pro440, Leu466, Ala467, Ile474, Phe486, Asp492, Ile493, Leu496, and Ile497 in Cavity 1, and with Gln123, Leu125, Tyr126, Tyr237, Phe238, Tyr239, Asp240, Phe241, Tyr242, Gln249, and Tyr252 in Cavity 2.

The binding affinity of phosphatidylcholine for β‐conglutin was significantly higher than the reported values for its affinities with ovalbumin (−0.096 kcal/mol) (Zhang et al. 2022), pea legumin, and vicilin (−6.91 and −7.04 kcal/mol) (Xia et al. 2024). This suggested that phosphatidylcholine actively interacted with β‐conglutin. On the basis of the number of poses, phosphatidylcholine showed greater compatibility with Cavity 1 than with Cavity 2 and this compatibility increased on raising the pH to 8.0. As phosphatidylcholine had similar affinity across cavities and pH values, this indicated that pH affected spatial availability for binding rather than affinity. Raising the pH expanded the area of negative electrostatic potential around Cavity 2, where the phosphatidylcholine heads were oriented at pH 6.0, prompting their reorientation at pH 7.0. This, combined with the gradual reduction in hydrophobic surface area at the region where the corresponding tails were oriented at pH 6.0 and 7.0, weakened hydrophobic interactions with the tails, resulting in the loss of binding poses at Cavity 2 at pH 8.0. Thus, the impact of the observed lecithin–protein complexation on protein behavior was subsequently characterized.

### 3.4 Characterization of Protein Dispersions Used to Prepare the Mimics

**FIGURE 4:**
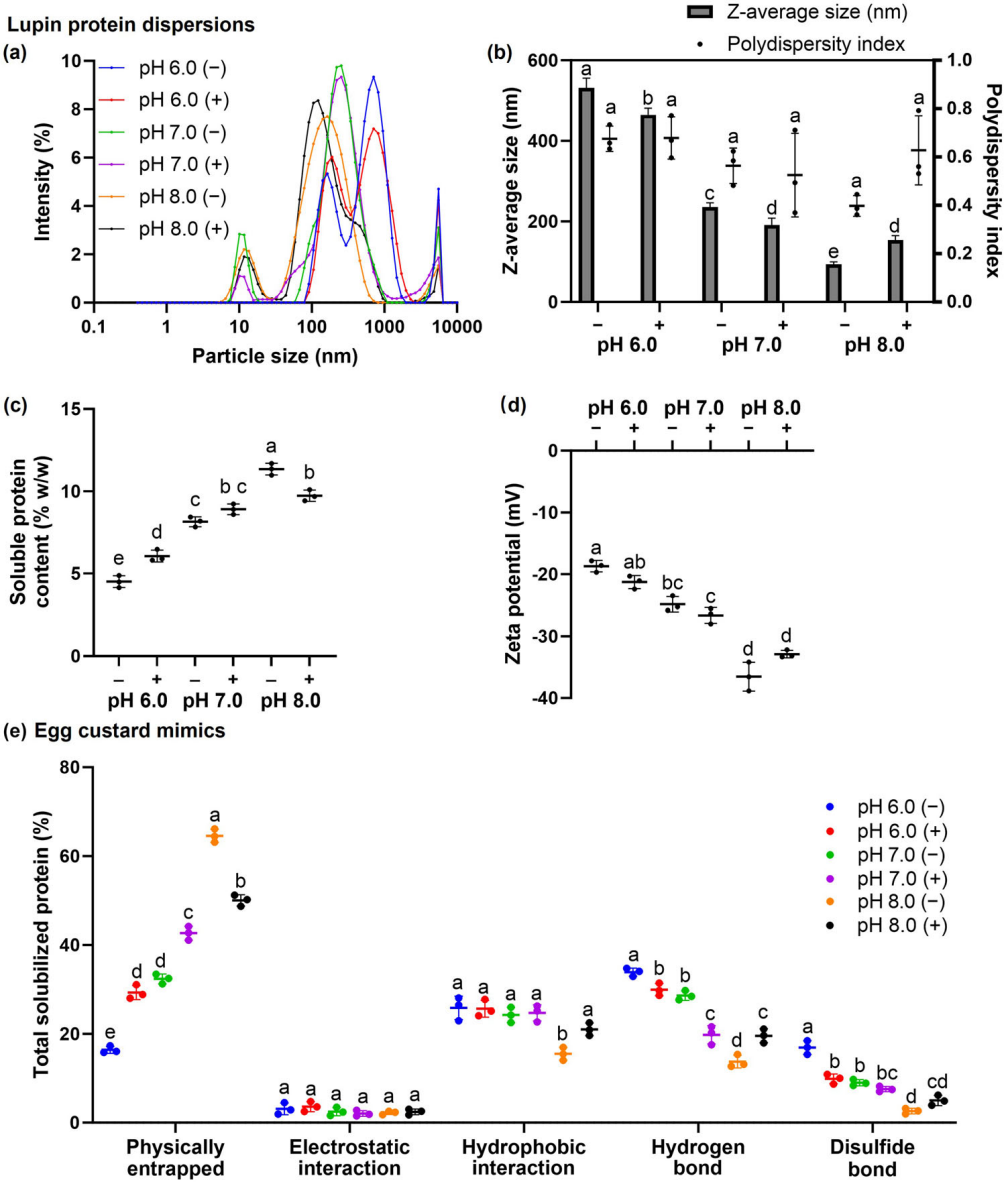
Protein structuring behavior evaluated at different pH levels, where (−) indicates the absence and (+) the presence of lecithin. For the dispersions: (a) shows the intensity‐based particle size distribution, (b) the Z‐average size, (c) the achievable soluble protein content, and (d) the zeta potential. For the corresponding egg custard mimics, (e) shows the proportion of protein stabilized by various molecular interactions. Different letters indicate significant differences (*p* < 0.05) among the mimics for each parameter in (b)–(d) and for each interaction in (e).

To analyze protein–lecithin aggregates, the lupin‐protein dispersions used to prepare the egg custard mimics were formulated without oil to eliminate potential interference (Xia et al. 2024). β‐Conglutin measured 16.663 nm × 16.663 nm × 4.010 nm based on x‐ray diffraction (Dolot et al. 2023), whereas α‐conglutin, the other major lupin protein, had a maximum width of approximately 17 nm based on AlphaFold predictions (UniProt ID: F5B8V7) (Varadi et al. 2024). Considering this, the 10.1–11.7 nm peak observed in the dispersions at pH 7.0 and 8.0 likely corresponded to free protein molecules (Figure 4a). The dominant peak shifted from 122.4 and 164.2 nm in the respective pH 8.0 dispersions to 255.0 nm in the pH 7.0 dispersions. This, coupled with the larger area under the curve between 1000 and 10,000 nm in the pH 7.0 dispersions compared to those at pH 8.0, indicated a higher extent of protein aggregation at pH 7.0. In comparison, the first peak in the pH 6.0 dispersions occurred at 164.2 and 190.1 nm, respectively, and the main peak was at 712.4 nm. Together with the highest peak intensities at 5560 nm, this suggested that protein aggregation was most extensive at pH 6.0, with a relative absence of free protein molecules. At this pH, the addition of lecithin increased the area under the smallest peak at the expense of the two larger peaks, appearing to reduce protein aggregation. These observations aligned with the changes in Z‐average size (Figure 4b). Raising the pH and adding lecithin at pH 6.0 and 7.0 significantly decreased the Z‐average size of the dispersions, whereas lecithin addition at pH 8.0 led to a significant increase (*p* < 0.05). Therefore, the Z‐average size was largest in the pH 6.0 (−) mimic at 531.6 nm and smallest in the pH 8.0 (−) mimic at 93.8 nm. The polydispersity index ranged from 0.40 to 0.68, consistent with the distributions being polydisperse.

As observed for β‐conglutin, raising the pH disrupted protein aggregation by increasing the negativity of the electrostatic surface potential. This reduced hydrophobic surface areas (Figure 3), thereby weakening hydrophobic interactions. Moreover, the resulting increase in electrostatic repulsion among proteins potentially disrupted hydrogen bonding (Liang et al. 2024). The decrease in these interactions likely drove the reduction in protein aggregation observed with increasing pH. Moreover, as the hydrophobic interactions and hydrogen bonds became less extensive at higher pH, the extent of their disruption through phospholipid–protein complexation was increasingly outweighed by the interactions that were promoted. The phospholipids promoted the interactions by associating with hydrophobic residues buried within the protein core—as observed in phosphatidylcholine–β‐conglutin interactions (Figure S3)—thereby exposing these residues at the hydrophilic surface. Similar conformational changes have been reported for pea, soy, and whey proteins in the presence of lecithin (Xia et al. 2024; Xiao et al. 2024; Pan et al. 2024). These exposed residues could then engage in hydrophobic interactions, including π–π stacking among tryptophan, phenylalanine, and tyrosine, which stabilize intermolecular β‐sheets and, in turn, support hydrogen bonding within these structures (Son et al. 2025; Tay et al. 2025). Lecithin addition thus promoted protein aggregation at pH 8.0 but disrupted it at pH 6.0 and 7.0, particularly since the consistent binding affinity of phosphatidylcholine for β‐conglutin across the pH range (Table S6) suggested similar extents of lecithin–protein complexation. This was schematically illustrated in Figure S4 and aligned with the hypothesis.

Reducing the pH of the pH 8.0 (−) dispersion, which corresponded to LS1, and adding lecithin both significantly lowered the achievable soluble protein content (*p* < 0.05) (Figure 4c). In contrast, lecithin addition at pH 6.0 and 7.0 increased the achievable soluble protein content in the dispersions. This tended to decrease as Z‐average size increased, likely due to larger protein aggregates being more prone to becoming insoluble. Additionally, as the pH of the dispersions increased, their zeta potential became increasingly negative (*p* < 0.05) (Figure 4d). The increase in zeta potential negativity with rising pH was consistent with the electrostatic surface potential changes of β‐conglutin (Figure 3). Adding lecithin slightly (*p* > 0.05) altered the zeta potential negativity, increasing it from −18.70 to −21.26 mV at pH 6.0 and from −24.85 to −26.64 mV at pH 7.0, while reducing it from −36.54 to −32.89 mV at pH 8.0. This was likely because lecithin addition at pH 6.0 and 7.0 caused protein disaggregation, which likely exposed shielded acidic residues within the aggregates (Pan et al. 2024). Correspondingly, at pH 8.0, lecithin promoted protein aggregation, which reduced the negativity of the zeta potential. Beyond particle size, the pH‐dependent changes in zeta potential could be attributed to the top‐scoring phosphatidylcholine poses on β‐conglutin localizing to both Cavities 1 and 2 at pH 6.0 and 7.0 but being confined to Cavity 1 at pH 8.0 (Figure 3). This shift in docking sites may have induced distinct conformational changes in β‐conglutin, which, as the dominant protein, influenced the exposure of acidic residues within the aggregates, thereby affecting surface charge (Xia et al. 2024; Xiao et al. 2024).

### 3.5 Molecular Interactions in the Mimics

The total proportion of protein solubilized from the gels of the egg custard mimics using the denaturants was similar (*p* > 0.05), ranging from 96.2% to 98.6%. Therefore, the proportion of protein released upon disruption of each interaction was representative of the proportion stabilized by that interaction (Xia et al. 2018). As the pH of the mimics without lecithin increased from 6.0 to 8.0, significant decreases (*p* < 0.05) were observed in the proportions of proteins held by hydrophobic interactions (from 25.8% to 15.5%), hydrogen bonding (from 33.9% to 13.7%), and disulfide bonding (from 16.9% to 2.6%) (Figure 4e). The reductions in hydrogen bonding and hydrophobic interactions aligned with the disruption of protein aggregation upon raising the pH, which was attributed to the increasing negativity of the electrostatic potential (Liang et al. 2024). The resulting increase in spatial separation of sulfhydryl groups in α‐conglutin (Figure 2) likely hindered their oxidation to disulfide bonds during heating.

On adding lecithin to the mimics, significant (*p* < 0.05) changes in the proportions of proteins held via various molecular interactions were observed. Specifically, the proportion of proteins held via hydrophobic interactions increased by 5.5% at pH 8.0. The proportion held via hydrogen bonding decreased by 4.0% at pH 6.0 and by 8.8% at pH 7.0 but increased by 5.8% at pH 8.0. The proportion held via disulfide bonding decreased by 7.1% at pH 6.0. These results largely aligned with the postulated changes in non‐covalent interactions—namely hydrophobic interactions and hydrogen bonding—that stemmed from lecithin's influence on protein aggregation. Upon raising the pH, the reduced extent of inherent non‐covalent interactions meant that their disruption through lecithin phospholipid–protein complexation was increasingly outweighed by newly formed interactions—both between lecithin and proteins and among proteins themselves (Tay et al. 2025; Xia et al. 2018). This likely accounted for the observed shifts in the proportion of proteins held via non‐covalent interactions across the different pH conditions. However, the absence of a decrease in the proportion of proteins held via hydrophobic interactions in the pH 6.0 (+) and pH 7.0 (+) mimics (*p* > 0.05) was likely due to hydrophobic interactions between the fatty acid chains of lecithin phospholipids and protein side chains offsetting the disruption of inherent ones (Xia et al. 2018). Additionally, the lecithin‐induced reduction in proteins held via hydrogen bonding in the pH 6.0 (+) mimic likely disrupted the proximity of sulfhydryl groups in α‐conglutin. This, in turn, could have hindered α‐conglutin—identified in Figure 2 as the primary contributor to disulfide bonding—from forming disulfide linkages due to impaired sulfhydryl oxidation (Zhou et al. 2020). Although lecithin influenced the extent of non‐covalent interactions as hypothesized, the resulting impact on disulfide bonding was observed only in the pH 6.0 mimic.

As raising the pH and adding lecithin to the pH 6.0 and 7.0 mimics reduced the proportions of proteins involved in intermolecular interactions, this resulted in corresponding increases in physically entrapped proteins, defined as those solubilized in the absence of denaturants (Tay et al. 2025). Conversely, lecithin addition to the pH 8.0 mimic decreased the proportion of physically entrapped proteins. As pH exerted a greater influence than lecithin on this proportion, it was lowest in the pH 6.0 (−) mimic at 16.4% and highest in the pH 8.0 (−) mimic at 64.6% (Figure 4e). Notably, this proportion increased by 32.2% between the pH 7.0 (−) and pH 8.0 (−) mimics—approximately double the increase observed between the pH 6.0 (−) and pH 7.0 (−) mimics—possibly due to a greater rise in zeta potential negativity (Figure 4d) and, thus, stronger electrostatic repulsion among protein aggregates.

### 3.6 SEM of the Mimics

**FIGURE 5:**
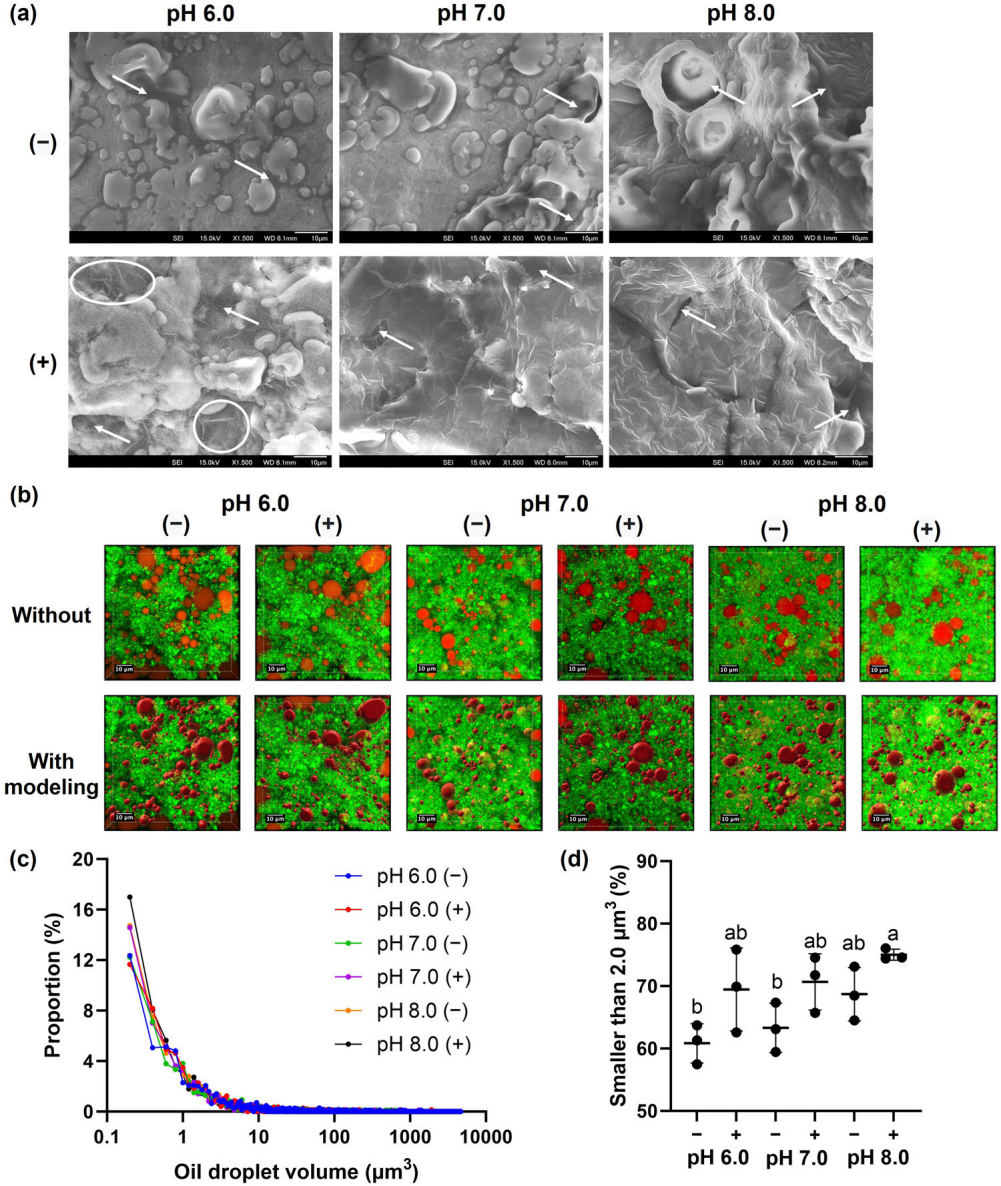
Microstructure of mimics at different pH levels, where (−) indicates the absence and (+) presence of lecithin. (a) SEM micrographs with 10 µm scale bars. White arrows indicate pores, and white circles in the pH 6.0 (+) mimic highlight regions altered by lecithin. (b) 3D CLSM images with 10 µm scale bars, presented with and without oil droplet modeling. CLSM‐based oil droplet volume analysis includes (c) the distribution and (d) the percentage of droplets smaller than 2.0 µm^3^. In (d), different letters indicate significant differences (*p* < 0.05) among the mimics.

Under SEM, the pH 8.0 (−) mimic exhibited wider and deeper pores than the pH 6.0 (−) and pH 7.0 (−) mimics (Figure 5a). Adding lecithin generally transformed the microstructure from an aggregated network of heterogeneous particles into one featuring continuous fine lines. This transformation was most pronounced in the pH 8.0 (+) mimic, where pores appeared smaller, and least pronounced in the pH 6.0 (+) mimic, where changes were confined to areas marked by white circles. Among mimics without lecithin, raising the pH disrupted protein interactions (Figure 4e) and weakened network connectivity, as reflected by the enlarged pore sizes at pH 8.0 (Liang et al. 2024). This likely reduced the network's resistance to structural alterations induced by lecithin. Consequently, the pH 6.0 (+) mimic retained regions resembling its counterpart without lecithin, likely consisting of lecithin‐complexed proteins that preserved inherent interactions to a relatively greater extent—possibly due to a lower degree of complexation—than the more altered regions (Zhou et al. 2020). In contrast, the microstructural connectivity of the pH 8.0 (+) mimic distinctly improved, consistent with its increased proportion of proteins held by intermolecular interactions (Figure 4d) (Tay et al. 2025).

### 3.7 Oil Droplet Volume Distribution in the Mimics Using CLSM

In the CLSM microstructures, the mimics exhibited oil droplets (red) dispersed throughout their protein networks (green) (Figure 5b). The majority of the oil droplets were smaller than 10.0 µm^3^ (Figure 5c). The proportion of droplets smaller than 2.0 µm^3^ increased with rising pH and the addition of lecithin, reaching 75.0% in the pH 8.0 (+) mimic, which was significantly higher than the 60.8%–63.3% observed in the pH 6.0 (−) and pH 7.0 (−) mimics (Figure 5d). This increase was attributed to protein aggregates becoming smaller and more negatively charged at higher pH (Figure 4), which facilitated the development of stronger viscoelastic films at the oil–water interface. These films enhanced electrostatic repulsion, thereby inhibiting oil droplet aggregation (Liu et al. 2024). Additionally, complexation of lecithin phospholipids with various proteins has been reported to increase their hydrophobicity and structural flexibility, including mung bean protein (Son et al. 2025), pea protein (Pan et al. 2024), and whey protein (Xia et al. 2024). Consequently, both phospholipid–protein complexes (Figure 3) and monomeric phospholipids likely coated the droplet surfaces more effectively than proteins alone (Son et al. 2025; Pan et al. 2024; Xia et al. 2024). The potential impact of oil droplet size on perceived greasiness was subsequently evaluated.

### 3.8 Analytical Gel Strength of the Mimics

**FIGURE 6:**
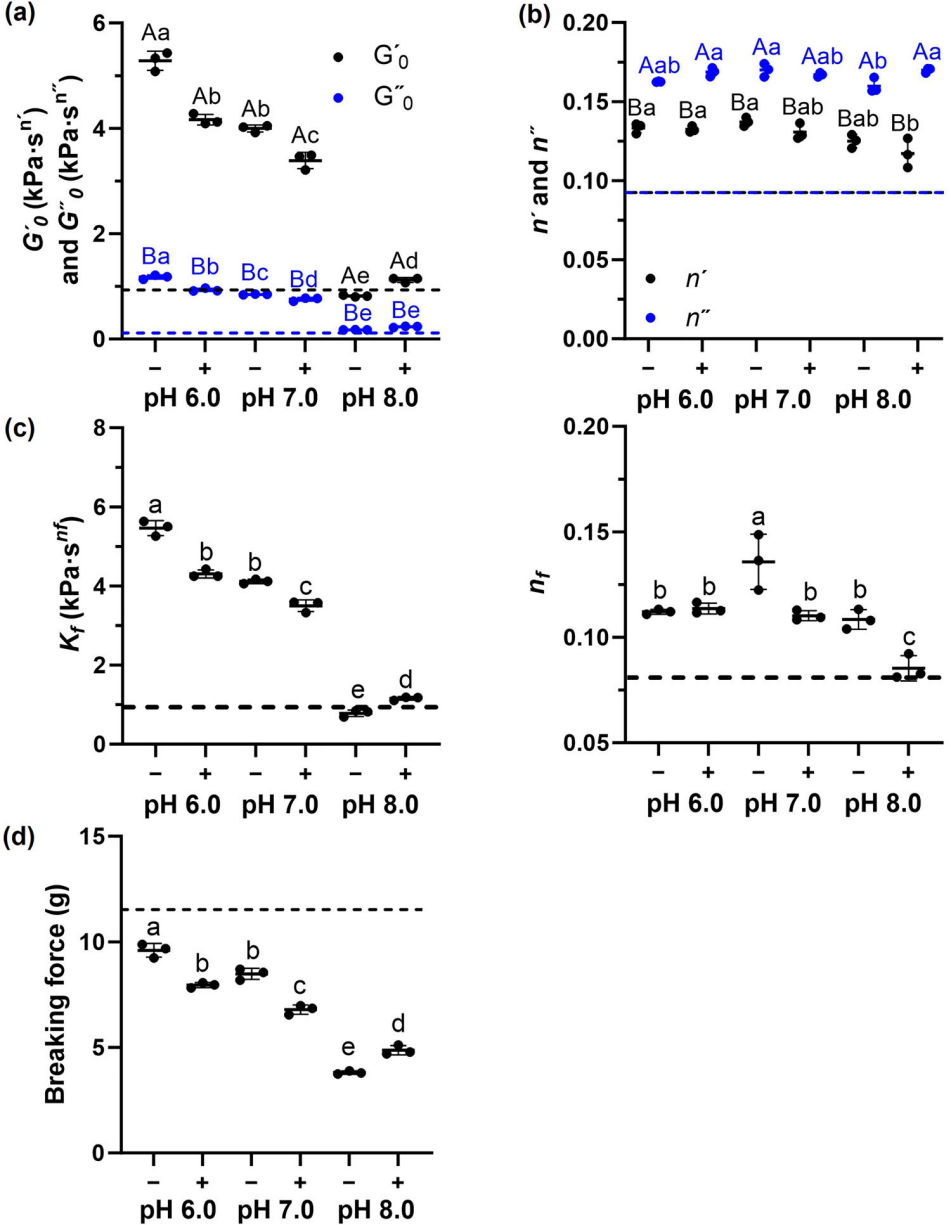
(a–c) Viscoelastic properties and (d) breaking force of mimics at different pH levels, where (−) indicates the absence and (+) indicates the presence of lecithin, evaluated relative to those of the control egg custard (dotted lines). In (a) *G′*_0_ and *G″*_0_, and (b) *n′* and *n″*, parameters modeled from the storage modulus *G′* are shown in black, while those modeled from the loss modulus *G″* are shown in blue. Part (c) comprises *K_f_* and *n_f_* values modeled from complex viscosity η*. Different lowercase letters indicate significant differences (*p* < 0.05) among the mimics for each parameter, whereas different uppercase letters indicate significant differences (*p* < 0.05) between *G′*_0_ and *G″*_0_, as well as between *n′* and *n″*, within each mimic.

The frequency sweeps of storage (*G′*) and loss (*G″*) moduli for the control egg custard and the egg custard mimics were measured at 0.5% strain, within the linear viscoelastic region where the sample structure was preserved (Figure S5a) (Lu et al. 2023). The *G′* of the mimics exceeded their respective *G″*, similar to that of the control egg custard (Figure S5b,c). This indicated that they were viscoelastic solids, with *G′* representing the elastic energy stored and *G″* representing the energy dissipated during deformation. This was also previously reported for lupin‐protein gels (Muranyi et al. 2016). The *G′* and *G″* were well‐fitted to Equations (1) and (2) with *R* ^2^ ≥ 0.901 and root mean square error (RMSE) ≤0.219 for *G′*, and *R* ^2^ ≥ 0.953 and RMSE ≤0.054 for *G″*. Modeling *G′* yielded *G′*_0_ and *n′*, whereas modeling *G″* yielded *G″*_0_ and *n″* (Figure 6a,b). Their complex viscosity η* (Figure S5d) was well‐fitted to Equation (3) (*R* ^2^ ≥ 0.988 and RMSE ≤0.140). Modeling η* yielded *K_f_* and *n_f_* (Figure 6c). Among both mimics with and without lecithin, increasing the pH from 6.0 to 8.0 significantly decreased *G′*_0_ (*p* < 0.05). Adding lecithin to the mimics decreased *G′*_0_ from 5.28 to 4.17 kPa s*^n^* at pH 6.0 and from 4.00 to 3.39 kPa s*^n^* at pH 7.0, while increasing it from 0.82 to 1.12 kPa s*^n^* at pH 8.0 (*p* < 0.05). As *G″*_0_ values followed similar trends to *G′*_0_ in response to pH and lecithin addition but were lower in magnitude, *K_f_* values were primarily influenced by *G′*_0_. Consistent with viscoelastic properties, increasing the pH of mimics with and without lecithin from 6.0 to 8.0 weakened the breaking force (*p* < 0.05) (Figure 6d). Adding lecithin to the mimics reduced the breaking force from 9.60 to 7.95 g at pH 6.0 and from 8.48 to 6.79 g at pH 7.0, while increasing it from 3.81 to 4.87 g at pH 8.0 (*p* < 0.05). Thus, lowering the pH of the mimics reinforced gel strength, whereas lecithin weakened it at pH 6.0 and 7.0 but enhanced it at pH 8.0.

In‐line with the hypothesis, the inherently lower extent of hydrophobic interactions and hydrogen bonding at pH 8.0 (Figure 4e) meant that their disruption by lecithin phospholipid–protein complexation was outweighed by newly formed interactions. This likely explained why lecithin increased the proportion of proteins held by these interactions in the pH 8.0 (+) mimic, while reducing the proportion held by hydrogen bonding at the lower pH values, namely, pH 6.0 (+) and pH 7.0 (+). In the pH 6.0 (+) mimic, the resulting loss of proximity among sulfhydryl groups in α‐conglutin was likely responsible for the observed decrease in disulfide bond formation (Zhou et al. 2020). Correspondingly, the pH 8.0 (+) mimic exhibited smaller pores that reinforced gel strength (Xia et al. 2018; Shi et al. 2020), whereas the pH 6.0 (+) and pH 7.0 (+) mimics showed weakened microstructural connectivity and, consequently, reduced gel strength (Zhou et al. 2020).

pH was found to have a greater impact than lecithin on the proportion of proteins involved in intermolecular interactions (Figure 4e), which, in turn, governed the gel strength of the mimics (Al‐Ali et al. 2021). The pH 6.0 (+) mimic exhibited a similar proportion of actively held proteins and thus comparable gel strength to the pH 7.0 (−) mimic. The pH 7.0 (+) mimic showed a lower proportion of actively held proteins and correspondingly reduced gel strength, although both values remained higher than those of the pH 8.0 (+) mimic (*p* < 0.05). Although reducing oil droplet size mitigated the disruption to network connectivity caused by droplets exceeding the pore size of the protein matrix (Wang et al. 2022), this did not enhance gel strength at higher pH, likely because the benefit was offset by weakened protein interactions. Similarly, the pH‐dependent effect of lecithin on gel strength suggested that lecithin primarily modulated gel strength by altering protein interactions (Tay et al. 2025). Otherwise, lecithin would have uniformly increased gel strength across pH if reduced droplet size were the dominant factor or uniformly decreased it if the weaker interaction of phospholipid‐stabilized droplets with the protein matrix—relative to protein‐stabilized ones—was the main influence (Lee et al. 2023; Torres et al. 2016).

The mechanical properties of the control egg custard did not clearly identify which mimic it most closely resembled. The mimics exhibited higher *n′* and *n″* values compared to the control egg custard (Figure 6b), indicating that their *G′* and *G″* were less temporally stable under deformation (Tay et al. 2025). Additionally, the *G′*, *G″*, and η* values of the control egg custard were as low as those of the pH 8.0 mimics (Figure S5), with *K_f_* and *n_f_* values further narrowing this similarity to the pH 8.0 (+) mimic (Figure 6c). However, the breaking force of the control egg custard exceeded those of the mimics (Figure 6d). Thus, QDA was performed to assess how these differences affected their perceived resemblance.

### 3.9 QDA of the Mimics

**FIGURE 7:**
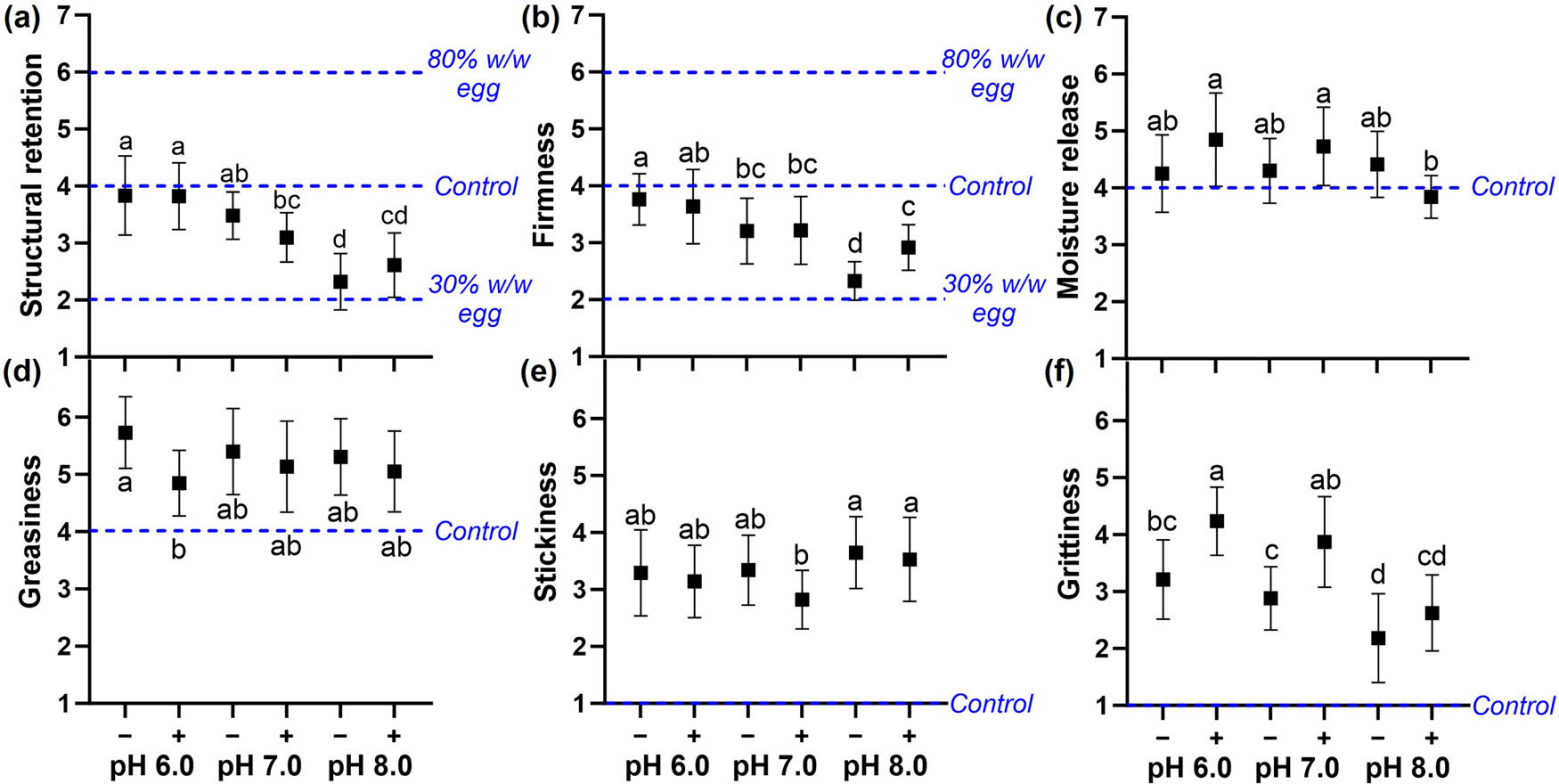
Perceived texture of mimics at different pH levels, where (−) indicates the absence and (+) the presence of lecithin. Dotted lines indicate values for the control egg custard and its variants. Different letters indicate significant differences (*p* < 0.05) among the mimics for each parameter. Parameters evaluated include (a) structural retention, (b) firmness, (c) moisture release, (d) greasiness, (e) stickiness, and (f) grittiness.

“Structural retention” assessed gel strength before mastication, whereas “firmness” assessed it during mastication (Table S4). The mimics exhibited intermediate structural retention, firmness, and consequently perceived gel strength between the control and soft egg custards, aligning more closely with breaking force than with viscoelasticity. Increasing the pH of both mimics, with and without lecithin, significantly decreased (*p* < 0.05) the perceived structural retention (Figure 7a) and firmness (Figure 7b). Moreover, adding lecithin to the mimic resulted in weaker structural retention at pH 7.0 and lower firmness at pH 6.0 (both *p* > 0.05), but enhanced structural retention (*p* > 0.05) and firmness (*p* < 0.05) at pH 8.0. These findings suggested that the effect of lecithin on analytical gel strength (Figure 6) extended to perceived gel strength, with both parameters largely influenced by pH. Additionally, the perceived “moisture release” of the mimics overlapped with that of the control egg custard and was distinctly higher than that of the Savoury Egg Mix gel, which was anchored at 2 (Figure 7c). This was likely because both the mimics and the control egg custard were protein‐based gels with carbohydrate‐to‐protein ratios of 0.07 to 0.26, distinctly lower than the 1.44 ratio of the Savoury Egg Mix gel (Table S3), which contained tapioca starch as its main ingredient.

The perceived “greasiness” of the mimics was within the typical range for egg custards, positioned between the control egg custard and a variant with added oil anchored at 7 (Figure 7d), likely due to their intermediate lipid content (Table S5). Adding lecithin to the mimic at pH 6.0 significantly decreased greasiness (*p* < 0.05). Slight reductions in greasiness were also observed with lecithin addition at other pH levels and with increasing pH in the absence of lecithin. These reductions corresponded to higher proportions of oil droplets smaller than 2.0 µm^3^ among the respective mimics (Figure 5d). Similarly, Wang et al. (2021) observed that oil droplet volume distribution influenced the perceived greasiness of emulsions. Although the pH 8.0 (+) mimic had the smallest oil droplets, the pH 6.0 (+) mimic was perceived as the least greasy (*p* > 0.05). This indicated that, besides the volume of oil droplets, gel strength and oil droplet clustering could influence greasiness perception (Fuhrmann et al. 2019).

The intermediate carbohydrate‐to‐protein ratio of the mimics (0.26), which falls between that of the Savoury Egg Mix gel (1.44) and the control egg custard (0.07) (Table S3), might explain their moderate “stickiness” (Figure 7e). Starch used in the Savoury Egg Mix gel tends to impart stickiness (Lu et al. 2023). Although no polysaccharides were added to the mimics, the inherent complexation of lupin protein with polysaccharides led to a higher carbohydrate content than that of the control egg custard (Table S3). Among the mimics, perceived stickiness increased slightly when the pH was raised to 8.0 (*p* > 0.05), as weakened gels tend to lose cohesiveness and become pasty (Lu et al. 2023).

The control egg custard was rated less “gritty” than the plant‐based samples, including both the mimics and the Myvegan Soy Protein Isolate emulsion gel (Figure 7f), likely due to its lower total solids content (6.5%) compared to the 12.4%–13.1% found in the plant‐based samples (Table S3). The mimics were rated distinctly lower in grittiness (2.6–4.2) than the Myvegan sample, which was anchored at 6. This corresponded with the markedly higher achievable soluble protein content in the dispersions used to prepare the mimics (4.51%–11.34% w/w), compared to that of the Myvegan Soy Protein Isolate (0.35% ± 0.03% w/w) (Figure 4c). This reflected a considerably higher soluble protein fraction in the former, given that the dispersions were standardized to 11.0% protein. Moreover, the reduction in grittiness observed in the mimics upon raising the pH—both with and without lecithin—was accompanied by an increase in achievable soluble protein content in the corresponding dispersions, reaching 9.73%–11.34% w/w at pH 8.0. This suggested that the mimics at pH 8.0 were formulated almost entirely from soluble proteins. The observed associations supported the postulation that insoluble proteins, being generally larger, had a greater tendency to impart grittiness. Higher grittiness was similarly reported in yogurt containing apple pomace powder with larger hydrated diameters (Grygorczyk and Blake 2023). Nonetheless, consumer acceptance tests are needed to determine whether the observed reduction in grittiness justifies the 4.7‐fold decrease in protein extraction yield from lupin flour resulting from the removal of insoluble proteins at pH 8.0 (Section 3.1). Notably, the soluble protein fraction could not explain the consistent rise in grittiness observed with lecithin addition across pH. Instead, this may be attributed to microstructural changes in the lecithin‐containing mimics (Figure 5a).

Texturally, the lecithin‐containing mimics exhibited gel strength, moisture release, and greasiness within the typical range of egg custards. Although they were perceived as grittier and stickier than the control egg custard, these attributes were less pronounced than in the respective commercial samples. This textural resemblance highlights their market potential, further supported by protein and phospholipid contents comparable to those of egg custard (Table S3). However, the impact of the lecithin‐induced increase in grittiness on consumer acceptability remains to be evaluated. Taken together, realizing the considerable potential of the lecithin‐containing mimics as a nutritious alternative that meets the growing demand for sustainable, animal‐free egg substitutes and accommodates individuals with egg allergies (Khalifa et al. 2025) will require addressing the challenges outlined.

## 4 Conclusions

This article characterized the influence of lecithin on texture of lupin‐protein emulsion gels at various pH as egg custard mimics. As hypothesized, with hydrophobic interactions and hydrogen bonding among proteins being least extensive at the highest pH of 8.0, those disrupted by phospholipid complexation were outweighed by those it promoted—both among proteins and between proteins and phospholipids. Lecithin thus enhanced network connectivity and ultimately strengthened the pH 8.0 (+) mimic. In contrast, lecithin reduced hydrogen bonding in the pH 6.0 (+) and pH 7.0 (+) mimics, which also diminished disulfide bonding in the pH 6.0 (+) mimic, thereby weakening both mimics. In addition to modulating the effect of lecithin on the proportion of proteins retained through molecular interactions and, consequently, the strength of the mimic, pH had a greater influence on these parameters. Accordingly, the pH 6.0 (−) mimic was the strongest and the pH 8.0 (−) mimic the weakest, with intermediate strengths achievable through lecithin addition. The textural resemblance of the mimics—including perceived gel strength—to that of traditional egg custards highlights their practical value. However, to realize this value, it is necessary to address how grittiness affects consumer acceptability and how protein extraction yield affects production scalability. Furthermore, the dependence of lecithin's texturing effect on inherent protein interactions underscores the value of computational molecular simulations. Such simulations could help mechanistically characterize how lecithin modulates these interactions and predict whether practical lecithin concentrations could achieve targeted modulation in a given protein system. This would support the rational incorporation of lecithin into protein emulsion gels for plant‐based egg, meat, and seafood applications to optimize their stability and texture.

## Nomenclature

(−): absence of lecithin in the egg custard mimic
η*
: complex viscosity
(+): presence of lecithin in the egg custard mimic
3D: three‐dimensional
CLSM: confocal laser scanning microscopy
FITC: fluorescein‐5‐isothiocyanate
*G′*
: storage modulus
*G″*
: loss modulus
*K_f_*
: dynamic consistency index
LP1: lupin‐protein pellet 1
LP2: lupin‐protein pellet 2
LS1: lupin‐protein supernatant 1
LS2: lupin‐protein supernatant 2
*n_f_*
: flow behavior index
QDA: quantitative descriptive analysis
SDS–PAGE: sodium dodecyl sulfate–polyacrylamide gel electrophoresis
SEM: scanning electron microscopy

## Author Contributions

**Uma Jingxin Tay**: conceptualization, methodology, investigation, formal analysis, visualization, writing – original draft, writing – review and editing, validation. **Jun Wei Ng**: conceptualization, investigation, writing – original draft, methodology, visualization, writing – review and editing, formal analysis. **Shiyi Zhang**: methodology, investigation, formal analysis. **Daryl Lee**: investigation, methodology, formal analysis. **Chengxin He**: investigation, methodology, formal analysis. **Dingsong Lin**: methodology, investigation, formal analysis. **Paolo Alberto Lorenzini**: conceptualization, investigation, methodology, formal analysis, software, visualization, writing – original draft, writing – review and editing. **Maria N. Antipina**: funding acquisition, writing–review and editing, supervision, resources. **Weibiao Zhou**: funding acquisition, writing – review and editing, supervision, resources. **Dejian Huang**: conceptualization, funding acquisition, project administration, supervision, resources, writing – review and editing, writing – original draft, validation.

## Conflicts of Interest

The authors declare no conflicts of interest.

## Supporting information




**Supplementary Material**: jfds70479‐sup‐0001‐SuppMat.docx

## Data Availability

Data will be made available on request.

## References

[jfds70479-bib-0001] Al‐Ali, H. A. , U. Shah , M. J. Hackett , M. Gulzar , E. Karakyriakos , and S. K. Johnson . 2021. “Technological Strategies to Improve Gelation Properties of Legume Proteins With the Focus on Lupin.” Innovative Food Science & Emerging Technologies 68: 102634. 10.1016/j.ifset.2021.102634.

[jfds70479-bib-0002] American Oil Chemists' Society . 2017. “AOCS Standard Procedure Am 5‐04.” In Official Methods and Recommended Practices of the American Oil Chemists' Society. Edited by D. Firestone . American Oil Chemists' Society. pp. 1–3.

[jfds70479-bib-0002a] >Alghooneh, A. , S. M. A. Razavi , and F. Behrouzian . 2017. “Rheological characterization of hydrocolloids interaction: A case study on sage seed gum‐xanthan blends.” Food Hydrocolloids 66: 206–215. 10.1016/j.foodhyd.2016.11.022.

[jfds70479-bib-0003] AOAC . 2023. Official Methods of Analysis of AOAC International. Edited by G. W. Latimer, Jr. Oxford University Press. 10.1093/9780197610145.001.0001.

[jfds70479-bib-0004] Bushnell, C. , L. Specht , and J. Almy . 2024. 2023 State of the Industry Report. Good Food Institute. https://gfi.org/wp‐content/uploads/2024/05/2023‐State‐of‐the‐industry‐report_Plant‐based.pdf.

[jfds70479-bib-0005] Caudill, M. A. 2010. “Pre‐ and Postnatal Health: Evidence of Increased Choline Needs.” Journal of the American Dietetic Association 110, no. 8: 1198–1206. 10.1016/j.jada.2010.05.009. 20656095

[jfds70479-bib-0006] Czubinski, J. , and S. Feder . 2019. “Lupin Seeds Storage Protein Composition and Their Interactions With Native Flavonoids.” Journal of the Science of Food and Agriculture 99, no. 8: 4011–4018. 10.1002/jsfa.9627. 30723906

[jfds70479-bib-0007] Devkota, L. , K. Kyriakopoulou , D. Fernandez , R. Bergia , and S. Dhital . 2023. “Techno‐Functional and Rheological Characterisation of Protein Isolates From Two Australian Lupin Species as Affected by Processing Conditions.” International Journal of Food Science and Technology 59, no. 2: 774–784. 10.1111/ijfs.16832.

[jfds70479-bib-0008] Dolot, R. M. , O'Sullivan, C. K. , and Jauset‐Rubio, M. 2023. Crystal Structure of Beta‐Conglutin From *Lupinus albus* Refined to 2.81 A. Protein Data Bank. https://www.rcsb.org/structure/8OFD.

[jfds70479-bib-0009] Duranti, M. , A. Consonni , C. Magni , F. Sessa , and A. Scarafoni . 2008. “The Major Proteins of Lupin Seed: Characterisation and Molecular Properties for Use as Functional and Nutraceutical Ingredients.” Trends in Food Science & Technology 19, no. 12: 624–633. 10.1016/j.tifs.2008.07.002.

[jfds70479-bib-0010] Fontanari, G. G. , J. M. Martins , M. Kobelnik , et al. 2011. “Thermal Studies on Protein Isolates of White Lupin Seeds (*Lupinus albus*).” Journal of Thermal Analysis and Calorimetry 108, no. 1: 141–148. 10.1007/s10973-011-1898-6.

[jfds70479-bib-0011] Fuhrmann, P. L. , L. C. M. Kalisvaart , G. Sala , E. Scholten , and M. Stieger . 2019. “Clustering of Oil Droplets in O/W Emulsions Enhances Perception of Oil‐Related Sensory Attributes.” Food Hydrocolloids 97: 105215. 10.1016/j.foodhyd.2019.105215.

[jfds70479-bib-0012] Grygorczyk, A. , and A. Blake . 2023. “Particle Perception: Defining Sensory Thresholds for Grittiness of Upcycled Apple Pomace Powders.” Food Quality and Preference 111: 104985. 10.1016/j.foodqual.2023.104985.

[jfds70479-bib-0013] Hedayati, S. , and M. M. Tehrani . 2018. “Effect of Total Replacement of Egg by Soymilk and Lecithin on Physical Properties of Batter and Cake.” Food Science & Nutrition 6, no. 4: 1154–1161. 10.1002/fsn3.656. 29983980 PMC6021699

[jfds70479-bib-0014] Johansson, M. , S. Karkehabadi , D. P. Johansson , and M. Langton . 2023. “Gelation Behaviour and Gel Properties of the 7S and 11S Globulin Protein Fractions From Faba Bean (*Vicia faba* var. minor) at Different NaCl Concentrations.” Food Hydrocolloids 142: 108789. 10.1016/j.foodhyd.2023.108789.

[jfds70479-bib-0015] Khalifa, I. , Z. Li , A. Nawaz , et al. 2025. “Recent Innovations for Improving the Techno‐Functional Properties of Plant‐Based Egg Analogs and Egg‐Mimicking Products to Promote Their Industrialization and Commercialization.” Comprehensive Reviews in Food Science and Food Safety 24, no. 1: e70086. 10.1111/1541-4337.70086. 39674849 PMC11646226

[jfds70479-bib-0016] Kuhn, M. , S. Firth‐Clark , P. Tosco , A. Mey , M. Mackey , and J. Michel . 2020. “Assessment of Binding Affinity Via Alchemical Free‐Energy Calculations.” Journal of Chemical Information and Modeling 60, no. 6: 3120–3130. 10.1021/acs.jcim.0c00165. 32437145

[jfds70479-bib-0017] Lee, J. , M.‐J. Choi , and Y. L. Xiong . 2023. “Comparative Effects of Micro Vs. submicron Emulsions on Textural Properties of Myofibrillar Protein Composite Gels.” Food Structure 38: 100353. 10.1016/j.foostr.2023.100353.

[jfds70479-bib-0018] Liang, G. , Y. Wen , W. Chen , et al. 2024. “Enhancing Soy Protein Isolate Gels: Combined Control of pH and Surface Charge for Improved Structural Integrity and Gel Strength.” Food Bioscience 59: 103934. 10.1016/j.fbio.2024.103934.

[jfds70479-bib-0019] Liu, Q. , A. Chen , P. Hong , C. Zhou , X. Li , and M. Xie . 2024. “pH‐Induced Interface Protein Structure Changes to Adjust the Stability of Tilapia Protein Isolate Emulsion Prepared by High‐Pressure Homogenization.” Food Chemistry: X 24: 101841. 10.1016/j.fochx.2024.101841. 39377085 PMC11456911

[jfds70479-bib-0020] Lu, Z. , P.‐R. Lee , and H. Yang . 2023. “Using HPMC to Improve Sensory Properties of Vegan Omelet Analogue: Effect of HPMC on Water Retention, Oil Adsorption, and Thermal Gelation.” Food Hydrocolloids 144: 108938. 10.1016/j.foodhyd.2023.108938.

[jfds70479-bib-0021] Mariotti, F. , D. Tomé , and P. Mirand . 2008. “Converting Nitrogen Into Protein—Beyond 6.25 and Jones' Factors.” Critical Reviews in Food Science and Nutrition 48: 177–184. 10.1080/10408390701279749. 18274971

[jfds70479-bib-0022] Martínez‐Rosell, G. , T. Giorgino , and G. D. Fabritiis . 2017. “PlayMolecule ProteinPrepare: A Web Application for Protein Preparation for Molecular Dynamics Simulations.” Journal of Chemical Information and Modeling 57, no. 7: 1511–1516. 10.1021/acs.jcim.7b00190. 28594549

[jfds70479-bib-0023] McNutt, A. T. , P. Francoeur , R. Aggarwal , et al. 2021. “GNINA 1.0: Molecular Docking With Deep Learning.” Journal of Cheminformatics 13, no. 1: 43. 10.1186/s13321-021-00522-2. 34108002 PMC8191141

[jfds70479-bib-0024] Morais Ferreira, J. M. , B. M. Azevedo , V. Luccas , and H. M. Bolini . 2017. “Sensory Profile and Consumer Acceptability of Prebiotic White Chocolate With Sucrose Substitutes and the Addition of Goji Berry (*Lycium barbarum*).” Journal of Food Science 82, no. 3: 818–824. 10.1111/1750-3841.13632. 28181242

[jfds70479-bib-0025] Muranyi, I. S. , C. Otto , C. Pickardt , R. Osen , P. Koehler , and U. Schweiggert‐Weisz . 2016. “Influence of the Isolation Method on the Technofunctional Properties of Protein Isolates From *Lupinus angustifolius* L.” Journal of Food Science 81, no. 11: C2656–C2663. 10.1111/1750-3841.13515. 27706815

[jfds70479-bib-0026] Quiroga, A. V. , P. Aphalo , J. L. Ventureira , E. Nora Martínez , and M. C. Añón . 2012. “Physicochemical, Functional and Angiotensin Converting Enzyme Inhibitory Properties of Amaranth (*Amaranthus hypochondriacus*) 7S Globulin.” Journal of the Science of Food and Agriculture 92, no. 2: 397–403. 10.1002/jsfa.4590. 21834100

[jfds70479-bib-0027] Pan, Y. , L. Liu , J. Li , et al. 2024. “Enhancing the Physical Stability and Bioaccessibility of Curcumin Emulsions Through the Interaction of Whey Protein Isolate and Soybean Lecithin.” Food Bioscience 58: 103676. 10.1016/j.fbio.2024.103676.

[jfds70479-bib-0028] Sasaki, K. , G. Watanabe , M. Motoyama , et al. 2019. “Descriptive Sensory Traits of Cooked Eggs Laid From Hens Fed Rice Grain.” Journal of Poultry Science 56, no. 3: 231–235. 10.2141/jpsa.0180082. PMC7005385 32055219

[jfds70479-bib-0029] Shi, T. , G. Y. A. Wijaya , L. Yuan , et al. 2020. “Gel Properties of Amur Sturgeon (*Acipenser schrenckii*) Surimi Improved by Lecithin at Reduced and Regular‐Salt Concentrations.” RSC Advances 10, no. 51: 30896–30906. 10.1039/D0RA04487C. 35516014 PMC9056329

[jfds70479-bib-0030] Son, C.‐G. , G.‐P. Hong , and Y.‐J. Jo . 2025. “Fabrication and Stability of Oil‐in‐Water Emulsions With Mung Bean Protein Aggregates and Soy Lecithin.” Colloids and Surfaces A: Physicochemical and Engineering Aspects 705: 135661. 10.1016/j.colsurfa.2024.135661.

[jfds70479-bib-0031] Tay, U. J. , J. Y. H. Toy , C. He , et al. 2025. “Soy Lecithin Strengthens Amaranth Protein‐Based Omelettes Through Protein Complexation Which Alters Protein Interactions.” Food Structure 44: 100432. 10.1016/j.foostr.2025.100432.

[jfds70479-bib-0032] Torres, O. , B. Murray , and A. Sarkar . 2016. “Emulsion Microgel Particles: Novel Encapsulation Strategy for Lipophilic Molecules.” Trends in Food Science & Technology 55: 98–108. 10.1016/j.tifs.2016.07.006.

[jfds70479-bib-0033] Varadi, M. , D. Bertoni , P. Magana , et al. 2024. “AlphaFold Protein Structure Database in 2024: Providing Structure Coverage for Over 214 Million Protein Sequences.” Nucleic Acids Research 52, no. D1: D368–D375. 10.1093/nar/gkad1011. 37933859 PMC10767828

[jfds70479-bib-0034] Wang, Q. , Y. Zhu , Z. Ji , and J. Chen . 2021. “Lubrication and Sensory Properties of Emulsion Systems and Effects of Droplet Size Distribution.” Foods 10, no. 12: 3024. 10.3390/foods10123024. 34945575 PMC8700785

[jfds70479-bib-0035] Wang, Y. , J. Zhao , S. Zhang , et al. 2022. “Structural and Rheological Properties of Mung Bean Protein Emulsion as a Liquid Egg Substitute: the Effect of pH Shifting and Calcium.” Food Hydrocolloids 126: 107485. 10.1016/j.foodhyd.2022.107485.

[jfds70479-bib-0036] Xia, B. , Y. Shen , R. Zhao , J. Deng , and C. Wang . 2024. “Interactions With Soy Lecithin Regulate the Emulsification Capacity of Pea Protein: Effects of Soy Lecithin Concentration.” Food Hydrocolloids 155: 110168. 10.1016/j.foodhyd.2024.110168.

[jfds70479-bib-0037] Xia, W. , L. Ma , X. Chen , X. Li , and Y. Zhang . 2018. “Physicochemical and Structural Properties of Composite Gels Prepared With Myofibrillar Protein and Lecithin at Various Ionic Strengths.” Food Hydrocolloids 82: 135–143. 10.1016/j.foodhyd.2018.03.044.

[jfds70479-bib-0038] Xiao, Y. , M.‐H. Pan , Y.‐S. Chiou , et al. 2024. “Mechanistic Understanding of the Effects of Nanoliposome‐Soybean Protein Isolate Interactions on Soybean Protein Isolate Emulsifying Properties.” Food Structure 39: 100357. 10.1016/j.foostr.2023.100357.

[jfds70479-bib-0039] Zhang, T. , Y. Yang , M. Zhang , et al. 2022. “Effect of Soy Lecithin Concentration on Physiochemical Properties and Rehydration Behavior of Egg White Protein Powder: Role of Dry and Wet Mixing.” Journal of Food Engineering 328: 111062. 10.1016/j.jfoodeng.2022.111062.

[jfds70479-bib-0040] Zhou, X. , H. Lin , S. Zhu , X. Xu , F. Lyu , and Y. Ding . 2020. “Textural, Rheological and Chemical Properties of Surimi Nutritionally‐Enhanced With Lecithin.” LWT 122: 108984. 10.1016/j.lwt.2019.108984.

