# Supplementary material for: pH‐Dependent Effect of Lecithin on Lupin‐Protein‐Based Egg Custard Mimics
Source: J Food Sci. 2025 Aug 22;90(8):e70479. doi: 10.1111/1750-3841.70479 (PMC12374080; doi:10.1111/1750-3841.70479)
Supplement: Supplementary file 1 — Supplementary Material: jfds70479‐sup‐0001‐SuppMat.docx [file JFDS-90-0-s001.docx]

# Supplementary Material

## List of Supporting Figures

**Fig. S1.** Evaluation of LS1 emulsions containing 30% to 80% egg-equivalent content (w/w)—formulated at 10% intervals based on the protein and lipid contents of egg—for their ability to form gels upon steaming at (A) pH 6.0, (B) pH 7.0, and (C) pH 8.0.

**Fig. S2.** The back view of the protein surface and lecithin docking sites corresponding to a 180° horizontal and 90° vertical rotation. C1 denotes Cavity 1 and C2 denotes Cavity 2.

**Fig. S3.** 2D view showing the interactions between phosphatidylcholine and β-conglutin. Interactions are colored light and dark green to indicate hydrogen bonds, blue to indicate weak hydrogen bonds, purple to indicate electrostatic interactions, grey to indicate hydrophobic interactions, and orange to indicate steric clashes.

**Fig. S4.** Schematic illustration of the influence of lecithin and pH on protein aggregation. (−) indicates the absence and (+) the presence of lecithin. Hydrophobic regions of the proteins are shown in yellow, and the electrostatic potential of various protein regions is represented by their respective charges.

**Fig. S5.** Viscoelastic properties of mimics at different pH levels, where (−) indicates the absence and (+) the presence of lecithin, evaluated relative to those of the control egg custard. (a) Storage modulus *G′* and loss modulus *G"* as a function of shear strain: Dots (•) indicate values for *G′*, while triangles (▼) indicate values for *G".* (b) *G′*, (c) *G"*, and (d) complex viscosity *η** as a function of angular frequency.

## List of Supporting Tables

**Table S1.** Proximate composition (% w/w) of various ingredients, with values presented as mean ± standard deviation (n = 3).

**Table S2.** Proximate composition and ingredient composition (g 100 g⁻¹) of LS1 emulsions containing 30% to 80% egg-equivalent content (w/w), formulated at 10% intervals based on the protein and lipid contents of egg.

**Table S3.** Proximate composition and ingredient composition (g 100 g⁻¹) of the control liquid egg, emulsions used to prepare the mimics, and other dispersions used for Quantitative Descriptive Analysis. Ingredients not added are indicated by ‘0’. Values are presented as mean ± standard deviation (n = 3).

**Table S4.** Texture attributes and definitions alongside the screening sets and intensity references. Perceived intensity was rated on a continuous 7-point scale, with 1 indicating the mildest intensity and 7 the strongest.

**Table S5.** Intensity of LS1, LS2, LP1, and LP2 bands analyzed via reducing and non-reducing SDS-PAGE. Significant differences (*p* < 0.05) among band intensities are indicated by different letters.

**Table S6.** Binding affinity (kcal/mol) and CNN affinity of the top-scoring docking poses of lecithin on 7S globulin for Cavities 1 and 2 at pH 6.0, 7.0, and 8.0 are presented as mean $\pm$ standard deviation. Significant differences (*p* < 0.05) among the sample means for each parameter are represented by different letters.


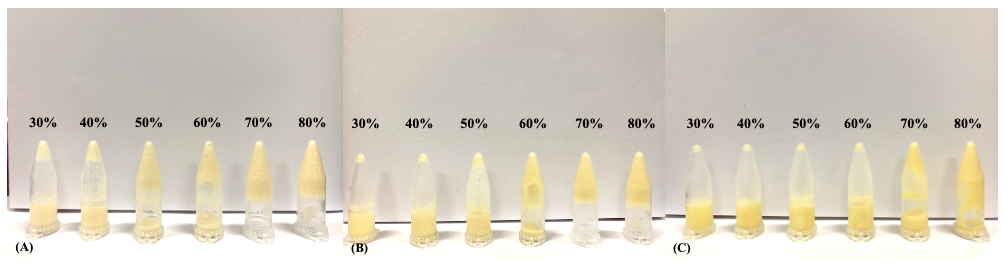


**Fig. S1.** Evaluation of LS1 emulsions containing 30% to 80% egg-equivalent content (w/w)—formulated at 10% intervals based on the protein and lipid contents of egg—for their ability to form gels upon steaming at (A) pH 6.0, (B) pH 7.0, and (C) pH 8.0.


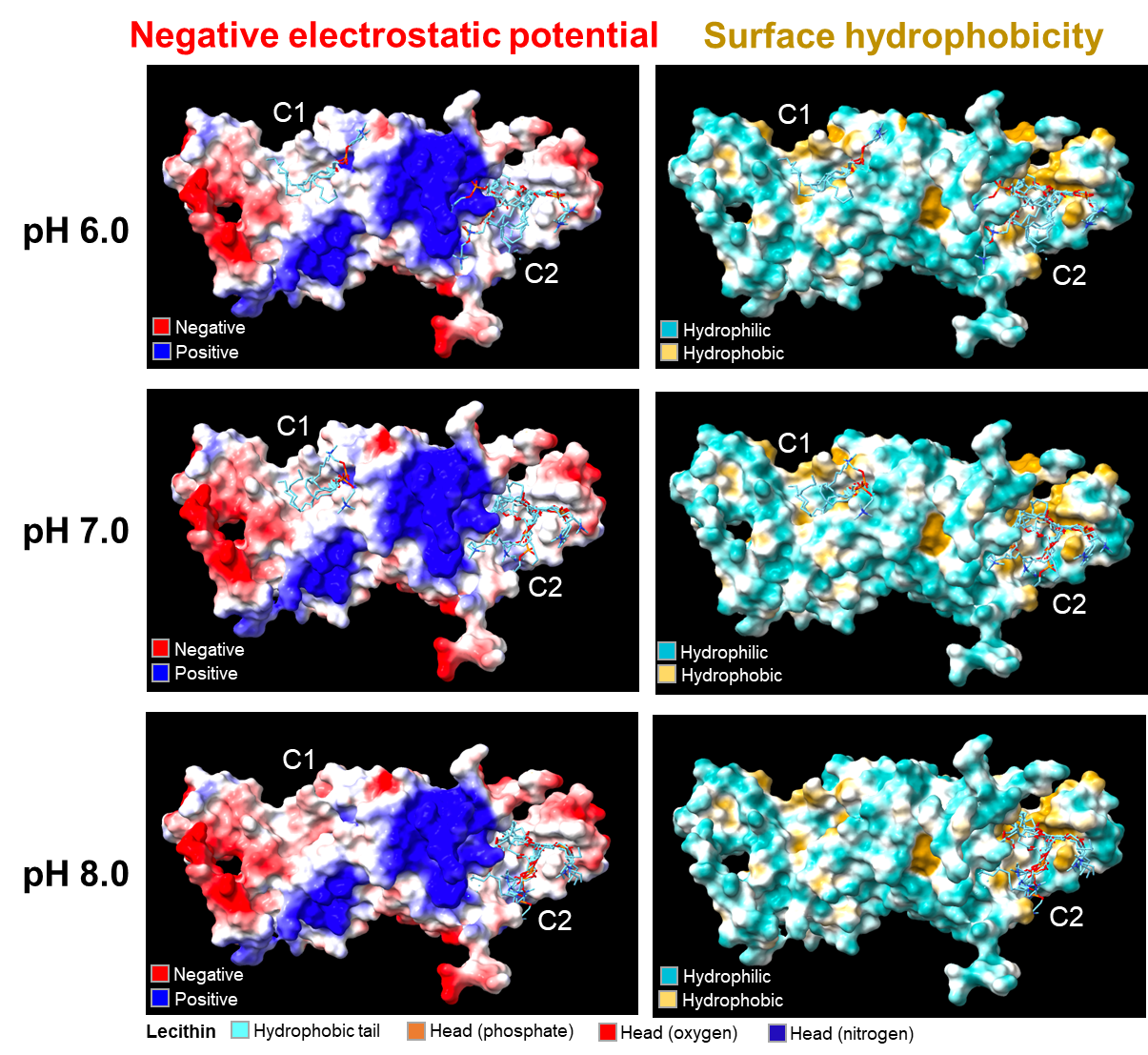


**Fig. S2.** The back view of the protein surface and lecithin docking sites corresponding to a 180° horizontal and 90° vertical rotation. C1 denotes Cavity 1 and C2 denotes Cavity 2.


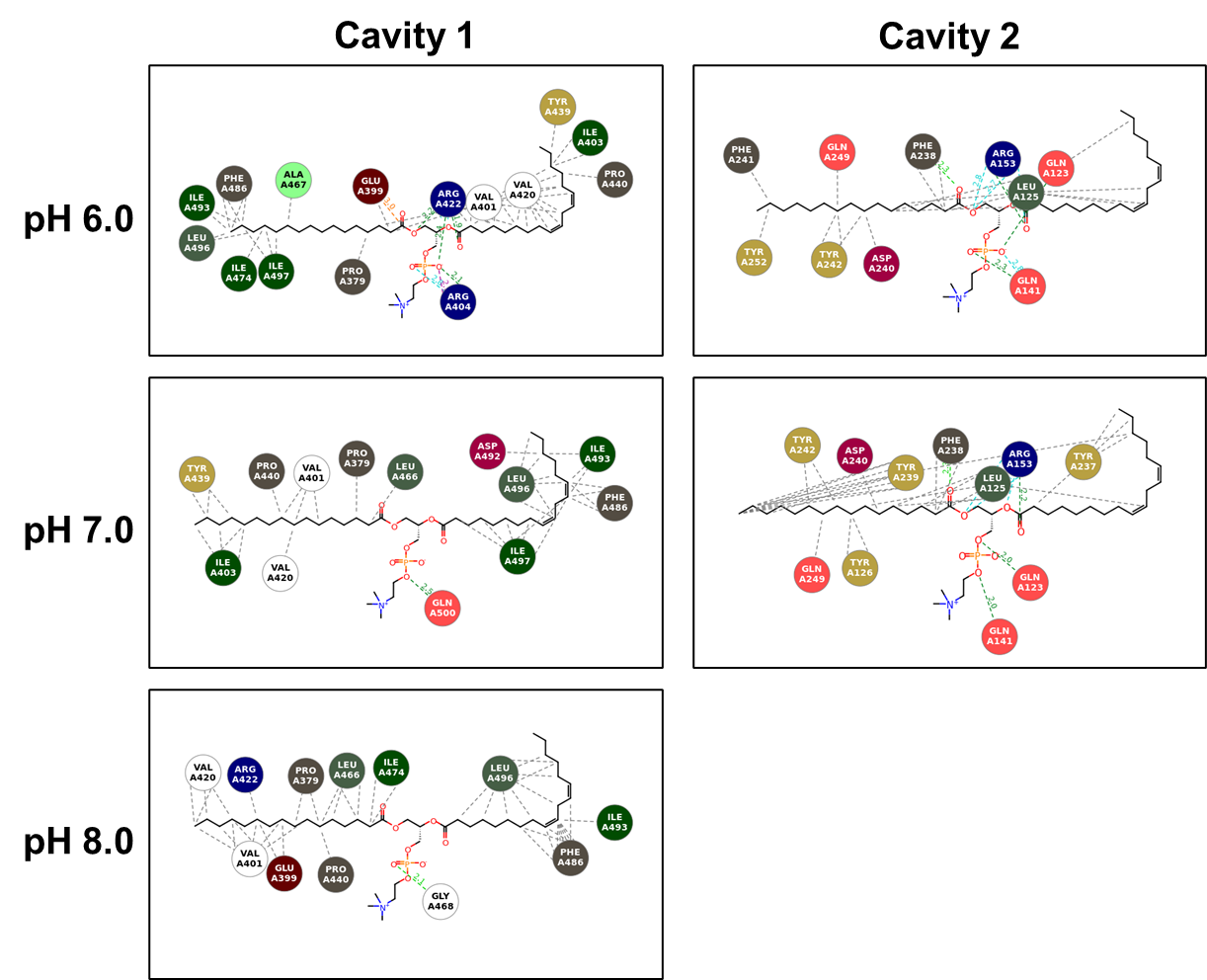


**Fig. S3.** 2D view showing the interactions between phosphatidylcholine and β-conglutin. Interactions are colored light and dark green to indicate hydrogen bonds, blue to indicate weak hydrogen bonds, purple to indicate electrostatic interactions, grey to indicate hydrophobic interactions, and orange to indicate steric clashes.


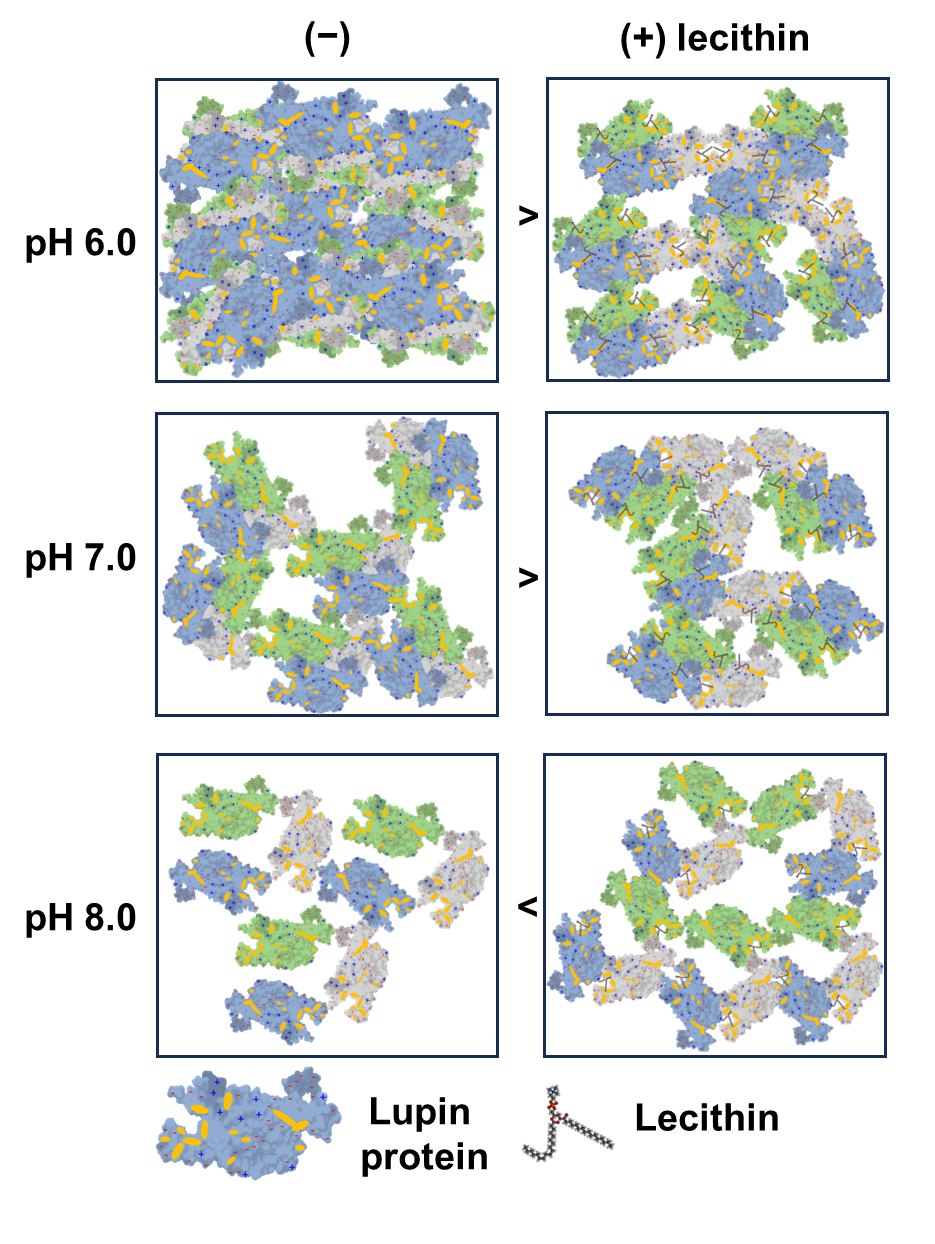


**Fig. S4.** Schematic illustration of the influence of lecithin and pH on protein aggregation. (−) indicates the absence and (+) the presence of lecithin. Hydrophobic regions of the proteins are shown in yellow, and the electrostatic potential of various protein regions is represented by their respective charges.


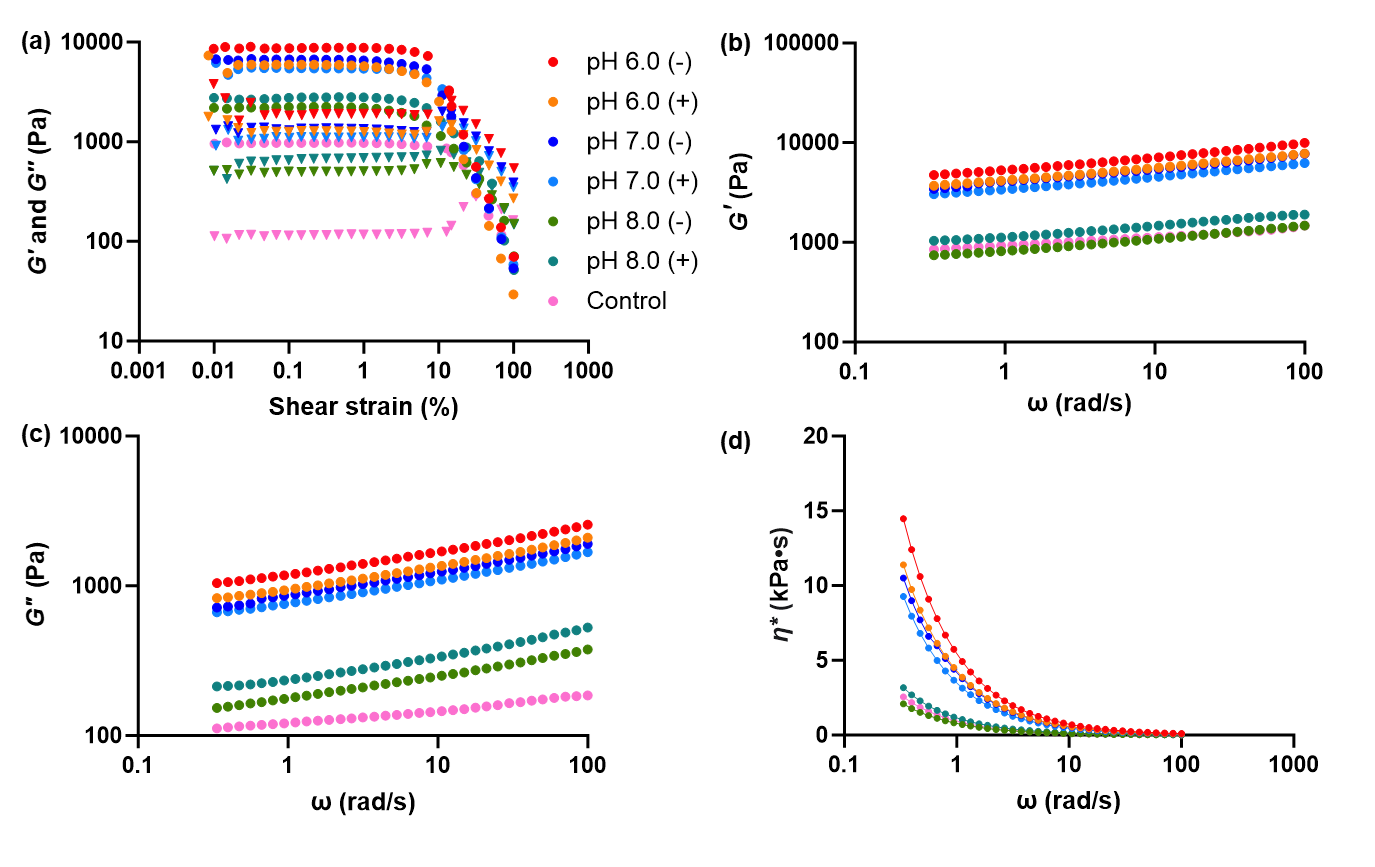


**Fig. S5.** Viscoelastic properties of mimics at different pH levels, where (−) indicates the absence and (+) the presence of lecithin, evaluated relative to those of the control egg custard. (a) Storage modulus *G′* and loss modulus *G"* as a function of shear strain: Dots (•) indicate values for *G′*, while triangles (▼) indicate values for *G".* (b) *G′*, (c) *G"*, and (d) complex viscosity *η** as a function of angular frequency.

**Table S1.** Proximate composition (% w/w) of various ingredients, with values presented as mean ± standard deviation (n = 3).

|  | Dry-basis | Wet-basis | | | |
| --- | --- | --- | --- | --- | --- |
|  | LS1^A^ | Hen egg | Soy lecithin | Savoury Egg Mix powder | Myvegan Soy Protein Isolate |
| Protein | 76.24 ± 1.21 | 12.41 ± 0.47 | 1.7^C^ | 22.47 ± 0.56^D^ | 73.92 ± 0.56 |
| Ash | 3.48 ± 0.30 | 1.12 ± 0.31 | 8.8^C^ | 5.94 ± 0.28 | 4.92 ± 0.32 |
| Lipid | 0.54 ± 0.10 | 9.96 ± 0.67 | 59.9^C^ | 28.70 ± 0.41 | 1.06 ± 0.45 |
| Phospholipid | N.A. | 1.0–3.0^B^ | 95.0^C^ | N.A. | N.A. |
| Carbohydrate | 19.75 ± 1.65 | 0.69 ± 0.10 | 3.7^C^ | 32.38 ± 0.93 | 13.68 ± 0.83 |
| Moisture | N.A.^B^ | 75.82 ± 0.40 | 1.6 ± 0.34 | 10.51 ± 0.47 | 6.42 ± 0.71 |

^A^ Standardized by expressing on a dry basis due to variable moisture content.

^B^ Hedayati and Mazaheri Tehrani (2018); Caudill (2010); Tay et al. (2025)

^C^ Determined from the Nutritional Information Sheet, accounting for moisture content.

^D^ A nitrogen-to-protein conversion factor of 5.4 was used, as it represents the average of the factors for lupin and pea proteins.

**Table S2.** Proximate composition and ingredient composition (g 100 g⁻¹) of LS1 emulsions containing 30% to 80% egg-equivalent content (w/w), formulated at 10% intervals based on the protein and lipid contents of egg.

|  | **30%** | **40%** | **50%** | **60%** | **70%** | **80%** |
| --- | --- | --- | --- | --- | --- | --- |
| Proximate composition (g 100 g⁻¹) | | | | | | |
| Protein | 3.72 | 4.96 | 6.21 | 7.45 | 8.69 | 9.93 |
| Ash | 0.17 | 0.23 | 0.28 | 0.34 | 0.4 | 0.45 |
| Lipid | 2.99 | 3.98 | 4.98 | 5.98 | 6.97 | 7.97 |
| Carbohydrate | 0.96 | 1.29 | 1.61 | 1.93 | 2.25 | 2.57 |
| Moisture | 92.15 | 89.54 | 86.92 | 84.31 | 81.69 | 79.08 |
| Ingredients (g 100 g⁻¹) | | | | | | |
| Deionized water, LS1 | 97.0 | 96.1 | 95.1 | 94.1 | 93.1 | 92.1 |
| Canola oil | 3.0 | 4.0 | 4.9 | 5.9 | 6.9 | 7.9 |

^A^ This refers to the phospholipids from soy lecithin.

^B^ The amount of deionized water added was determined by the moisture content of LS1, which was measured separately for each batch.**Table S3.** Proximate composition and ingredient composition (g 100 g⁻¹) of the control liquid egg, emulsions used to prepare the mimics, and other dispersions used for Quantitative Descriptive Analysis. Ingredients not added are indicated by ‘0’. Values are presented as mean ± standard deviation (n = 3).

|  | **Liquid egg** | | **Emulsions** | | **Savoury Egg Mix dispersion** | **Myvegan Soy Protein Isolate emulsion** |
| --- | --- | --- | --- | --- | --- | --- |
|  | **Control** | **Oil-added variant** | **Without lecithin** | **With lecithin** |  |  |
| ***Proximate composition*** | | | | | | |
| **Protein** | 5.58 | 4.46 | 9.92 | 9.94 | 5.95 | 9.94 |
| **Ash** | 0.50 | 0.40 | 0.45 | 0.54 | 1.57 | 0.66 |
| **Lipid** | 4.38 | 23.5 | 7.97 | 8.57 | 7.60 | 8.03 |
| ***From:*** |  |  |  |  |  |  |
| **egg** | 4.38 | 3.50 | N.A. | N.A. | N.A. | N.A. |
| **canola oil** | N.A. | 20.0 | 7.90 | 7.90 | N.A. | 7.90 |
| **soy lecithin** | N.A. | N.A. | N.A. | 0.63 | N.A. | N.A. |
| **LS1 or others** | N.A. | N.A. | 0.07 | 0.07 | 7.60 | 0.13 |
| **Phospholipid^A^** | 0.5–1.4 | 0.4–1.1 | N.A. | 0.95 | N.A. | N.A. |
| **Carbohydrate** | 0.41 | 0.33 | 2.57 | 2.61 | 8.58 | 1.84 |
| **Moisture** | 88.9 | 71.1 | 78.6 | 78.6 | 76.3 | 79.5 |
| ***Ingredient composition*** | | | | | | |
| **Egg** | 45.0 | 36.0 | 0 | 0 | 0 | 0 |
| **Deionized water** | 55.0 | 44.0 | 92.1^B^ | 91.1^B^ | 73.5 | 78.7 |
| **LS1 or other ingredients** | 0 | 0 |  |  | 26.5 | 13.4 |
| **Canola oil** | 0 | 20.0 | 7.90 | 7.90 | 0 | 7.90 |
| **Lecithin** | 0 |  | 0 | 1.00 | 0 |  |

^A^ This refers to the phospholipids from soy lecithin.

^B^ The amount of deionized water added was determined by the moisture content of LS1, which was measured separately for each batch.

^C^Ingredients that were not included are indicated by ‘0’.

**Table S4.** Texture attributes and definitions alongside the screening sets and intensity references. Perceived intensity was rated on a continuous 7-point scale, with 1 indicating the mildest intensity and 7 the strongest.

| **Order** | **Attributes** | **Definition** | **Intensity references** |
| --- | --- | --- | --- |
| 1 | Structural retention | The tendency of the egg custard to flow or disintegrate when loaded into the mouth before mastication. | 2: Egg custard prepared by steaming egg diluted to 30% w/w.  4: Control egg custard  6: Egg custard prepared by steaming egg diluted to 80% w/w. |
| 2 | Firmness | The perceived mastication force. |  |
| 3 | Moisture release | The extent to which a thin, low viscosity liquid was released on mastication. | 2: Savoury Egg Mix gel.  4: Control egg custard  7: A particulate dispersion prepared by steaming egg diluted to 15% w/w. |
| 4 | Greasiness | The perception of an oily and lubricating coating in the mouth, as opposed to a rough sensation. | 2: Myvegan Soy Protein Isolate emulsion gel.  4: Control egg custard  7: Oil-added egg custard variant |
| 5 | Stickiness | The adherence to various parts of the mouth including the tongue, palate and teeth. | 1: Control egg custard  7: Savoury Egg Mix gel.  4: Hybrid custard prepared by combining the control liquid egg with the Savoury Egg Mix dispersion at a 1:1 w/w ratio. |
| 6 | Grittiness | The perception of particles when rubbing the tongue against the palate. | 1: Control egg custard  6: Myvegan Soy Protein Isolate emulsion gel.  5: Hybrid custard prepared by combining the control liquid egg with the Myvegan Soy Protein Isolate emulsion at a 1:1 w/w ratio. |

**Table S5.** Intensity of LS1, LS2, LP1, and LP2 bands analyzed via reducing and non-reducing SDS-PAGE. Significant differences (*p* < 0.05) among band intensities are indicated by different letters.

|  | **LS1** | **LS2** | **LP1** | **LP2** |
| --- | --- | --- | --- | --- |
| 18–20 kDa | | | | |
| Non-reducing | 2.69 ± 0.39 ^a^ | 2.61 ± 0.05^a^ | 1.80 ± 0.10^b^ | 1.33 ± 0.07^b^ |
| Reducing | 1.59 ± 0.48^a^ | 1.92 ± 0.06^a^ | 1.81 ± 0.15^a^ | 1.48 ± 0.12^a^ |
| Cumulative 51.0–64.2 kDa | | | | |
| Non-reducing | 0.47 ± 0.02^a^ | 0.26 ± 0.02^b^ | 0.16 ± 0.02^c^ | 0.04 ± 0.02^d^ |
| Reducing | 0.26 ± 0.01^a^ | 0.19 ± 0.01^b^ | 0.16 ± 0.01^c^ | 0.07 ± 0.01^d^ |
| 52.3 kDa | | | | |
| Reducing | 0.17 ± 0.06^a^ | 0.18 ± 0.03^a^ | 0.37 ± 0.02^b^ | 0.37 ± 0.04^b^ |
| Cumulative 75.4–96.6 kDa | | | | |
| Reducing | Not detectable | 0.20 ± 0.03^a^ | 0.38 ± 0.03^b^ | 0.37 ± 0.04^b^ |
| Residual band which did not enter the gel | | | | |
| Non-reducing | 2.69 ± 0.39 ^a^ | 2.61 ± 0.05^b^ | 1.80 ± 0.10^c^ | 1.33 ± 0.07^c^ |

**Table S6.** Binding affinity (kcal/mol) and CNN affinity of the top-scoring docking poses of lecithin on 7S globulin for Cavities 1 and 2 at pH 6.0, 7.0, and 8.0 are presented as mean $\pm$ standard deviation. Significant differences (*p* < 0.05) among the sample means for each parameter are represented by different letters.

|  |  | **Binding affinity (kcal/mol)** | **CNN affinity** |
| --- | --- | --- | --- |
| pH 6.0 | C1 | -15.06 ± 0.42^a^ | 5.63 ± 0.22^a^ |
|  | C2 | -15.53 ± 0.20^a^ | 5.96 ± 0.11^a^ |
| pH 7.0 | C1 | -14.89 ± 0.37^a^ | 5.86 ± 0.40^a^ |
|  | C2 | -15.23 ± 0.16^a^ | 5.94 ± 0.46^a^ |
| pH 8.0 | C1 | -14.95 ± 0.34^a^ | 5.80 ± 0.19^a^ |
